# Cost-effectiveness of apixaban compared to other anticoagulants in patients with atrial fibrillation in the real-world and trial settings

**DOI:** 10.1371/journal.pone.0222658

**Published:** 2019-09-17

**Authors:** Lisa A. de Jong, Jessie Groeneveld, Jelena Stevanovic, Harrie Rila, Robert G. Tieleman, Menno V. Huisman, Maarten J. Postma, Marinus van Hulst

**Affiliations:** 1 Department of PharmacoTherapy, -Epidemiology & -Economics, University of Groningen, Groningen Research Institute of Pharmacy (GRIP), Groningen, the Netherlands; 2 Bristol Myers Squibb, Utrecht, the Netherlands; 3 Department of Cardiology, Martini Hospital, Groningen, the Netherlands; 4 Department of Cardiology, University of Groningen, University Medical Center Groningen, Groningen, the Netherlands; 5 Department of Thrombosis and Hemostasis, Leiden University Medical Centre, Leiden, the Netherlands; 6 Department of Economics, Econometrics & Finance, University of Groningen, Faculty of Economics & Business, Groningen, the Netherlands; 7 Department of Health Sciences, University of Groningen, University Medical Centre Groningen, Groningen, the Netherlands; 8 Department of Clinical Pharmacy and Toxicology, Martini Hospital, Groningen, The Netherlands; Inselspital Universitatsspital Bern, SWITZERLAND

## Abstract

**Introduction:**

Randomized clinical trials (RCTs) and real-world data (RWD) in patients with atrial fibrillation have shown that—compared to vitamin K antagonists (VKAs)—non-VKA oral anticoagulants (NOACs) are at least as effective in the prevention of ischaemic stroke, while decreasing the risk of bleeding.

**Objective:**

We aim to evaluate the cost-effectiveness of the NOAC apixaban versus other NOACs (dabigatran, edoxaban and rivaroxaban) and VKA, for stroke prevention in patients with atrial fibrillation by including the available data both from RCT and real-world analyses of all NOACs into one integrative previously published model.

**Methods:**

The model was updated to the current Dutch healthcare situation. The incremental cost-effectiveness ratio was calculated using either efficacy/effectiveness and safety data derived from a network meta-analysis (NMA) synthesizing NOAC RCTs or RWD. We conducted a systematic literature search to identify eligible publication to best inform the RWD-based analysis. Additional sensitivity and scenario analyses were conducted to test the robustness of the outcomes.

**Results:**

In the NMA-based analysis, apixaban appeared to be cost-effective compared to VKA (€3,506 per quality adjusted life-year) and dominant (cost-saving and more effective) over dabigatran 110 mg, dabigatran 150 mg, edoxaban and rivaroxaban. In the RWD-based analysis, apixaban was dominant over all other anticoagulants. In the scenario analysis apixaban appeared to be not cost-effective compared to dabigatran 150 mg, when using equal event-unrelated treatment discontinuation rates for each drug. In all other scenarios apixaban is cost-effective or cost-saving compared to VKA and other NOACs.

**Conclusion:**

Based on RCTs as well as RWD, we conclude that apixaban is generally cost-effective or even cost-saving (less costly and more effective) compared to VKA and other NOACs in the overall population of patients with atrial fibrillation.

## Introduction

Atrial fibrillation (AF) is the most common cardiac arrhythmia and is a major cause of ischemic stroke, heart failure and cardiovascular morbidity [1]. In the Netherlands, the prevalence in the total population is 2–3% [2] and in people of 75 years and older the prevalence of AF increases to more than 10% [3]. Furthermore, also due to the aging population, the number of patients with AF is predicted to rise steeply in the coming years [1].

AF constitutes a significant public health problem, and estimates suggest that this condition accounts for 1.3% of the national healthcare budget in the Netherlands with a total estimated cost of €583 million in 2009 [4]. In patients with AF, the associated increased risks of ischaemic stroke and systemic embolism (SE) present a major challenge. Approximately 20–30% of the patients experiencing ischaemic stroke have been diagnosed with AF before, during or after the stroke event [1]. Embolic events, such as ischaemic stroke, lead to high hospitalization costs and long-term maintenance costs which contributes to the high economic burden to the Dutch health care system [5].

In patients with AF, anticoagulant (AC) therapy with vitamin K antagonists (VKAs) has been the most effective treatment for the prevention of stroke over the last decades. However, on the basis of randomized clinical trial (RCT) results, the European guidelines have adopted the non-vitamin K antagonist oral anticoagulants (NOACs) as the preferred treatment for the prevention of stroke in patients with AF since 2012 [1,6]. A network meta-analysis (NMA) on pivotal RCTs in treatment groups with NOACs versus the VKA warfarin included data on almost 72,000 patients [7], showed that NOACs were at least as effective in the prevention of ischaemic stroke compared to VKA while there was a 50% decrease in the incidence of haemorrhagic stroke. Over the last years, real-world data (RWD) on the effectiveness and safety of NOACs have been published, confirming the results of the RCTs.

In terms of effectiveness, safety and costs it is relevant to investigate whether the results of the cost-effectiveness using RWD support results of analyses based on RCTs (external validation). Multiple cost-effectiveness analyses have been published on the different NOACs versus VKAs on the basis of the respective RCTs results, using different models and cost assumptions. Here, we aim to evaluate the cost-effectiveness of the NOAC apixaban versus other NOACs and VKAs for stroke prevention in patients with AF by including the available data both from NMA and real-world analyses of all available NOACs in one integrative previously published model [8].

## Methods

We aim to evaluate the cost-effectiveness of apixaban compared to VKA, dabigatran, rivaroxaban and edoxaban, by capturing all costs and health outcomes related to the disease, treatment and complications, during the lifetime of a patient with AF. The primary outcome of our analysis is the incremental cost-effectiveness ratio (ICER), calculated by dividing the incremental costs by the incremental health outcomes, presented in life-years LY and quality adjusted life-years (QALY). The ICER is compared to a willingness-to-pay (WTP) threshold of €20,000/QALY. This threshold is based on the disease burden calculation (proportional shortfall estimate of 0.14) in the calculation tool as recommended by the Dutch guidelines for pharmacoeconomic research [9]. The ICER will be calculated using either efficacy/effectiveness and safety data derived from an NMA synthesizing RCTs on NOACs [10] (NMA-based analysis) or RWD (RWD-based analysis).

We conducted a systematic literature search to identify eligible publication to best inform the RWD-based analysis. The systematic literature search was conducted with help of the ‘Preferred Reporting Items for Systematic Reviews and Meta-Analyses: The PRISMA Statement’, to identify RWD studies eligible for the RWD-based analysis [11]. Details on the method as well as the results of the search strategy are described in S1. Following the pre-defined eligibility criteria, the real-world study of Lip et al. [12] was considered the most appropriate for use in the RWD-based analysis. From this real-world study we obtained patient characteristics and real-world event rates to populate the RWD-based model. The model structure and all other input parameters were equal for both analyses.

### Model structure

**Fig 1:**
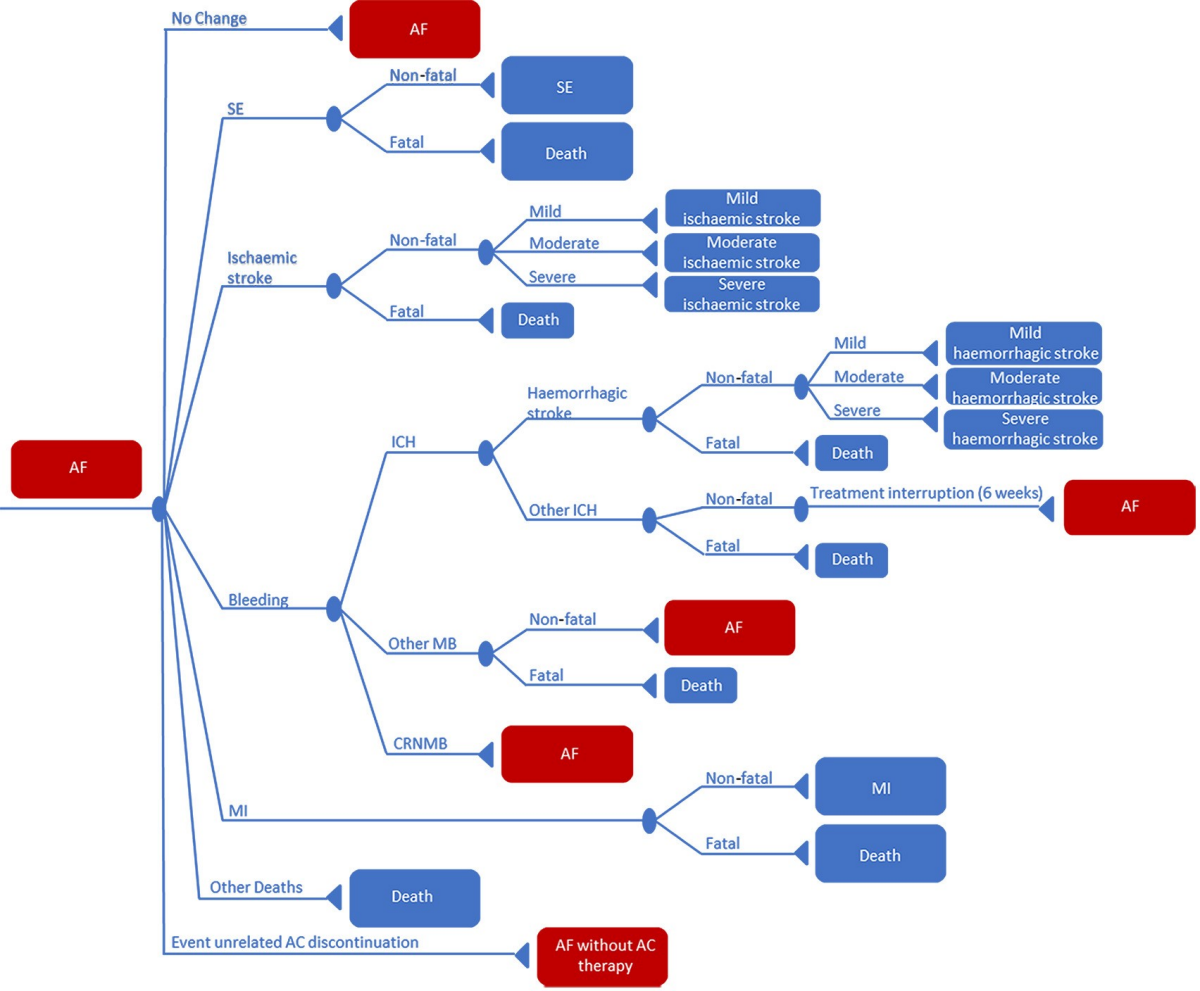
Representation of the Markov model. All patients entered the model in the AF state, from where they could move to another health state upon the occurrence of one of the following events: ‘ischaemic stroke’, ‘haemorrhagic stroke’, ‘other ICH’, ‘other MB’, ‘CRNMB’, ‘SE’, ‘MI’, ‘other deaths’ or ‘event unrelated AC discontinuation’. The triangles show the state a patient enters after an event. These states can be transient (red squares) or absorbing (blue squares). Abbreviations: AC, anticoagulant; AF, atrial fibrillation; CRNMB, clinically relevant non-major bleeding; ICH, intracranial haemorrhage; MB, major bleeding; MI, myocardial infarction; SE, systemic embolism.

Both the NMA-based and the RWD-based analyses were based on a previously published Markov model [8]. The model was validated and updated with data based on the most recent literature to evaluate the lifetime costs and health effects of treatment with apixaban compared to VKA and other NOACs in AF patients in the Netherlands. Since this model was designed with apixaban as the reference drug, indirect comparison between other NOACs (dabigatran, edoxaban and rivaroxaban) and comparison of other NOACs to VKA, was not possible [8]. To model the disease course of AF, different health states were included in the model. During a lifetime, a hypothetical cohort of 1,000 AF patients could remain in or move through these different health states. The risk to move to another health state, hereafter mentioned as the transition probability, was calculated per six weeks cycle. This cycle length was chosen to capture all events related to AF within such a short time frame [8]. As shown in Fig 1, the model included the following health states: ‘AF’, ‘ischaemic stroke’ (including unspecified strokes), ‘SE’, ‘bleeding’, myocardial infarction (‘MI’), ‘other deaths’ (all deaths unrelated to the aforementioned events) and ‘event unrelated AC discontinuation’ (AC discontinuation unrelated to the events explicitly included). Bleeding events were divided into intracranial haemorrhage (referred to as ‘ICH’, which was assumed to be the composite of ‘haemorrhagic stroke’ and ‘other ICH’), other major bleeding (referred to as ‘other MB’, defined as all non-ICH MBs) and clinically relevant non-major bleeding (‘CRNMB’). Additionally, a distinction was made between different levels of ischaemic and haemorrhagic stroke severity (i.e. mild, moderate and severe).

All patients entered the model in the ‘AF’ state. Upon the occurrence of a clinical event the patient remained in the corresponding health state for one cycle and then moved to one of the transient or absorbing states. ‘AF’ and ‘AF without AC therapy’ (red squares) reflect transient states, meaning that patients were able to remain in them or move to other states in the model. In absorbing states (e.g. ‘death’ [blue squares]) patients remained until death. Upon the occurrence of ‘event unrelated AC discontinuation’ patients were assumed to discontinue AC therapy permanently.

### Patient characteristics

In the NMA-based and RWD-based analyses, all patients entered the model with baseline characteristics as presented in S1 Table. In the NMA-based analysis, we used a Dutch study [13] to simulate specific Dutch patient characteristics, adapting the NMA to the specific Dutch context. The patients entered the model aged 72, 64.7% were male and the average CHADS_2_ score and CHA_2_DS_2_-VASc score were 1.7 and 3.1, respectively. In the RWD-based analysis, the patient characteristics were based on the selected real-world study itself. The patients were on average 74.3 years old, 54.1% were male and the average CHA_2_DS_2_-VASc score was 3.7.

### Transition probabilities

#### Event rates

In the NMA-based analysis, transition probabilities were based on the clinical event risks in patients receiving apixaban and a VKA (warfarin) in the ARISTOTLE trial [14]. Hazard ratios compared to apixaban for the comparators dabigatran (110 mg and 150 mg), rivaroxaban and edoxaban were obtained from an NMA by Lip et al [10], who made pairwise indirect comparisons between the ARISTOTLE [14], RE-LY [15], ROCKET-AF [16] and ENGAGE-AF [17] trials, using propensity score adjustments. S2 Table shows the event rates per 100 patient-years (PY) for apixaban and VKA and hazard ratios for dabigatran 110 mg, dabigatran 150 mg, rivaroxaban and edoxaban. Lip et al. also included edoxaban 30 mg in his analysis, however this low-dose was not approved by the European Medicines Agency and therefore not included in our analysis. Average stroke and bleeding (ICH and other MB) risks were weighted by multiplying the CHADS_2_ score distribution in Dutch patients (S1 Table) with the corresponding event risks (S2 Table). Subsequently, the event rates were calculated per six weeks (42 days) cycle [18]. Event rates for the other NOACs (dabigatran 110 mg, dabigatran 150 mg, rivaroxaban and edoxaban) were based on the event rate of apixaban per cycle multiplied by the hazard ratios (S2 Appendix) [18]. ‘Ischaemic stroke’ and ‘haemorrhagic stroke’ health states were specified per severity, by the modified Rankin Scale (mRS), specifying mild (mRS 0–2), moderate (mRS 3–4), severe (mRS 5) and fatal, based on RCT data [14–17]. The model allowed for one recurring ischaemic or haemorrhagic stroke, with an event rate of 2.72 per 100 PY, which was assumed to be equal across all treatments [19].

The transition probabilities used in the RWD-based analysis are summarized in S3 Table. Based on the real-world study by Lip et al. [12] we included RWD-based event rates of apixaban and VKA and hazard ratios of dabigatran and rivaroxaban for ischaemic stroke, ICH, other MB and SE, and distributions of haemorrhagic stroke among ICH and GI bleeding among other MB. If transition probabilities were unavailable from RWD, we used the same transition probabilities as used in the NMA-based analysis.

In sensitivity analysis, transition probabilities, hazard ratios and distributions were varied with a beta, log normal and Dirichlet distributions, respectively.

#### Mortality

Mortality rates are summarized in S4 Table. Background mortality was calculated by fitting the Gompertz distribution to 2017 Dutch life tables to obtain a proper representation of the mortality risk for each six weeks cycle [20]. For each health state, background mortality risks were updated by case fatality and mortality risk adjustment factors by using data from the ARISTOTLE trial [14] within the trial period and data from a Danish study by Brønnum et al. [21] for long term outcomes.

#### Treatment switch or discontinuation

Treatment discontinuation could be related or unrelated to an event. Upon the occurrence of non-fatal other ICH, patients were assumed to discontinue AC therapy for six weeks after which they resumed their original AC [22]. Patients who experienced a non-fatal haemorrhagic stroke or non-fatal MI were assumed to discontinue their AC therapy permanently and were assigned acute and long-term maintenance costs. All patients experiencing any other event such as ischaemic stroke or SE were assumed to continue their current AC therapy.

In case of ‘event unrelated AC discontinuation’, all event risks (e.g. event risk, case fatality, severity distributions) were updated to no treatment. These event rates for patients receiving no treatment, summarized in S5 Table, were obtained from a meta-analysis of placebo, aspirin and warfarin controlled studies in AF [23]. After AC discontinuation we assumed no further bleeding risk, and a constant risk of ischaemic stroke, SE, MI, and other CV hospitalisation, independent of time, prior AC therapy or patient characteristics.

### Utilities

In absence of health-state-specific EQ-5D Dutch utilities, health-state-specific utility scores were obtained from a UK EQ-5D catalogue by Sullivan et al. [24] (Table 1), similar to the previously published UK model [8]. A specific score for AF patients was used and updated upon the occurrence of a clinical event. Stroke (ischaemic and haemorrhagic) utility scores were subdivided per severity. Utility decrements for other ICH, other MBs, CRNMB, other CV hospitalization and use of AC therapy were taken into account [25,26]. Health outcomes were discounted at a rate of 1.5% per year [27]. Utility (decrement) parameters were varied using beta distributions for sensitivity analyses.

**Table 1:** Utilities for different health states.

**Utility Value**			
	**Value, mean (standard error)**		**Source**
AF	0.7270 (0.1818)		[24]
Ischaemic stroke			
Mild	0.6151 (0.1538)		[24]
Moderate	0.5646 (0.1412)		[24]
Severe	0.5142 (0.1285)		[24]
Haemorrhagic stroke			
Mild	0.6151 (0.1538)		[24]
Moderate	0.5646 (0.1412)		[24]
Severe	0.5142 (0.1285)		[24]
MI	0.6098 (0.1525)		[24]
SE	0.6265 (0.1566)		[24]
**Utility Decrement**			
	**Value, mean (standard error or 95% CI)**	**Duration**	**Source**
Other ICH (6 weeks)	0.1385 (0.0346)	6 weeks	[25]
Other MB (2 weeks)	0.1385 (0.0346)	14 days	[25]
CRNMB (2 days)	0.0600 (0.0150)	2 days	[25]
Other CV hospitalization (6 days)	0.1276 (0.0319)	6 days	[24]
Treatment with VKA	0.0130 (0.00–0.08)	While receiving treatment	[26]
Treatment with apixaban, dabigatran, rivaroxaban or edoxaban ^a^	0.0020 (0.00–0.04)	While receiving treatment	[26]

^a^ Utility decrement related to anticoagulation with NOACs is assumed to be equal to aspirin.

Abbreviations: AF, atrial fibrillation; CI, confidence interval; CRNMB, clinically relevant non-major bleeding; CV, cardiovascular; ICH, intracranial haemorrhage; MB, major bleeding; MI, myocardial infarction; SE, systemic embolism, VKA, vitamin K antagonist.

### Costs

Direct and indirect costs in- and outside the healthcare sector were considered, since the ICERs were calculated from a societal perspective, as recommended by the Dutch cost manual for pharmacoeconomic evaluation [27]. All costs with their corresponding ranges are summarized in Table 2. Ranges were based on a 95% confidence interval (CI). If 95% CI was unavailable, ranges were calculated with a standard error of 25% of the mean. Although this approach has limitations, as discussed in ISPOR-SMDM modelling good research practices report [28], the exclusion of parameters from a sensitivity analysis based on the fact that there is no data available to estimate probabilistic parameters would be less appropriate. Drug costs were derived from the offxicial Dutch price list (Z-index) from July 2018 [29]. The drug cost of VKA was based on the weighted average cost of phenprocoumon (21,1%) and acenocoumarol (78,9%) as reported in the annual medical report of the Dutch Federation of Thrombosis Service (FNT) [2]. For VKA patients the cost of an INR monitoring visit was calculated as the average cost of INR measurement at thrombotic service (45.6%), at home (34.0%) and self-measurement (20.4%) [27,30]. Based on the FNT medical report an average frequency of INR monitoring visits was set at 22.3 per year. Routine care for NOACs consisted of an annual general practitioner (GP) visit, since the European Society for Cardiology guidelines mention that chronic care management can be handled by GP [1]. AC management consisted of an additional GP visit for AC related dyspepsia and renal function monitoring, which should for NOACs be done at least once per year according to international guidelines [31].

**Table 2:** Drug costs, event costs and indirect costs for the different health states.

**Drug costs**			
**Drug**	**Average daily cost**		**Source**
Apixaban (5 mg BID)	€2.18		[29]
VKA (3 mg daily)	€0.05 ^a^		[29]
Dabigatran (110 mg BID)	€2.44		[29]
Dabigatran (150 mg BID)	€2.44		[29]
Rivaroxaban (20 mg daily)	€2.29		[29]
Edoxaban (60 mg daily)	€2.39		[29]
**Monitoring and routine care costs**			
**Unit**	**Cost per unit (range)**		**Source**
INR monitoring visit (VKA)	€15.03 €8.59 - €23.24) ^b^		[30]
Routine care related to AF (NOAC)	€33.76 (€19.30 - €52.21) ^c^		[27]
AC management			
Dyspepsia related GP visit	€33.76 (€19.30 - €52.21) ^c^		[27]
Renal monitoring	€6.69 (€3.82 - €10.35)		[27]
**Event costs**			
**Event**	**Acute care cost (range)**	**Long-term maintenance cost per month (range)**	**Source**
Ischaemic or haemorrhagic stroke			
Mild	€15,623 (€11,367 - €21,473)	€202 (€147- €278)	[25]
Moderate	€38,595 (€28,081 - €53,046)	€1,507 (€1,096 - €2,071)	[25]
Severe	€46,877 (€34,107 - €64,428)	€3,087 (€1,401 - €2,646)	[25]
Fatal	€3,088 (€1,765 - €4,774)		[25]
Other ICH	€21,004 (€15,283 - €28,869)		[25]
Other MB	€5,162 (€3,756 - €7,095)		[25]
CRNMB	€31.73 (€23 - €43)		[25]
MI	€5,189 (€5,101 - €5,276)	€202.54 (€189 - €213)	[25]
SE	€5,538 (€3,165 - €8,563)	€202.54 (€189 - €213)	[25]
Other CV hospitalization	€1,657 (€947 - €2,562) ^d^		[32]
**Indirect costs**			
**Unit**	**Acute care cost (range)**	**Long-term maintenance cost per month (range)**	**Source**
**Travel costs**			
INR monitoring visit (VKA)	€0.08 (€0.05 - €0.13)		[27]
Routine care related to AF (NOAC)	€0.21 (€0.12 - €0.32)		[27]
AC management	€0.21 (€0.12 - €0.32)		[27]
**Informal care costs**			
Ischaemic or haemorrhagic stroke			
Mild		€1,065 (€609 - €1,646)	[34]
Moderate		€1,407 (€804 - €2,176)	[34]
Severe		€1,750 (€1,000 - €2,705)	[34]
MI		€497 (€284 - €768)	[27,30]
SE		€497 (€284 - €768)	[27,30]

^a^ Based on weighted average of phenprocoumon and acenocoumarol users in the Netherlands

^b^ Assumption: weighted average of costs based on declaration codes for measurement at thrombotic service (DOT: 079995), at home (DOT: 079995 and 079986) and self-measurement/management (DOT: 190253)

^c^ Based on cost of one GP visit

^d^ Average of Dutch declaration codes for hospitalization of maximal 5 days for heart failure (DOT: 099899089), infection in the heart (DOT: 099899013) and high blood pressure (DOT: 090301003).

Abbreviations: AC, anticoagulant; AF, atrial fibrillation; BID, twice daily; CRNMB, clinically relevant non-major bleeding; CV, cardiovascular; GI, gastro-intestinal; GP, general practitioner; ICH, intracranial haemorrhage; INR, international normalized ratio; MB, major bleed; MI, myocardial infarct; NOAC, non-vitamin K antagonist oral anticoagulant; SE, systemic embolism; VKA, vitamin K antagonist.

Event costs per health state were divided into acute and long-term maintenance costs. Acute costs were related to hospital and rehabilitation facility costs and maintenance costs reflected medical costs during the patient’s lifetime [18]. Event costs were derived from our previous Dutch economic study [5]. Costs for other CV hospitalizations were based on Dutch hospital tariffs for hospitalization related to heart failure, high blood pressure and heart infections [32].

Indirect costs included travel expenses and event related informal care costs. Travel costs were included for 45% of the INR monitoring visits (patients who visited thrombosis service themselves) and for each routine care or dyspepsia related GP visits [33]. These costs were based on an average distance to the GP of 1.1 km, with a cost of €0.19 per km [27]. Informal care costs were added to other long-term maintenance costs following events with absorbing states. Informal care costs for stroke (ischaemic and haemorrhagic) were based on the Dutch costing study by van den Berg et al. (based on a mean of 29.4 hours of informal care per week) [34]. MI and SE were assumed to require less intensive informal care compared to stroke, and were therefore based on a Dutch report that defined non-intensive informal care as eight hours per week. The price per hour (€14) was derived from the Dutch cost manual for pharmacoeconomic evaluation [27].

All costs were inflated to 2018 using the consumer price index from Dutch Statistics (CBS Statline) [35]. Cost outcomes were discounted at a rate of 4% per year [27]. For sensitivity analyses the costs parameters were varied over a gamma distribution.

### Sensitivity and scenario analyses

Next to base case analyses based on the NMA and RWD, sensitivity analyses were conducted to evaluate the influence of uncertainty in input parameters on the ICER. In the probabilistic sensitivity analysis (PSA) costs, utilities, transition probabilities were varied simultaneously over their 95% CIs and ICERs were calculated during 2,000 simulations. Outcomes were presented in cost-effectiveness planes and cost-effectiveness acceptability curves. In the univariate sensitivity analysis input parameters were varied one by one over their 95% CI, to examine the influence on the ICER for each parameter separately. The input parameters with the highest impact on the ICER were presented in a Tornado diagram. Three additional scenario analyses were conducted to capture the effect of healthcare payer’s perspective (scenario 1), equal drug prices for all NOACs (scenario 2) and equal event unrelated AC discontinuation rates for all NOACs (scenario 3).

## Results

### Deterministic results

Table 3 summarizes the costs outcomes per category. Event costs are the largest contributor to the total costs (45–49% and 47–53% in the NMA-based and RWD-based analyses, respectively). Indirect costs also have high impact on the total costs: in both analyses 39–45% of the total costs are related to indirect costs. In VKA treated patients, the impact of drug costs is negligible compared to NOACs (<1%% vs. 8–10% of total costs). Overall relatively small differences in total costs were observed across different types of NOACs. The total costs of AF with NOACs as well as VKA treatment are lower in real-world setting compared to the trial setting.

**Table 3:** Base-case costs outcomes of the NMA-based and RWD-based analyses presented as costs per patient over a lifetime horizon.

**NMA-based analysis**						
	**Apixaban**	**VKA**	**Dabigatran 110 mg**	**Dabigatran 150 mg**	**Rivaroxaban**	**Edoxaban**
Drug costs	€ 3,925 (10%)	€ 95 (<1%)	€ 3,426 (8%)	€ 3,323 (8%)	€ 3,683 (9%)	€ 4,020 (10%)
Monitoring/ management costs	€ 1,181 (3%)	€ 2,192 (5%)	€ 1,148 (3%)	€ 1,179 (3%)	€ 1,174 (3%)	€ 1,176 (3%)
Event costs	€ 18,573 (45%)	€ 19,872 (49%)	€ 20,227 (46%)	€ 19,320 (46%)	€ 19,100 (46%)	€ 18,470 (45%)
Indirect costs	€ 17,289 (42%)	€ 18,005 (45%)	€ 18,811 (43%)	€ 17,905 (43%)	€ 18,010 (43%)	€ 17,463 (42%)
*Total costs*	€ 40,968	€ 40,163	€ 43,612	€ 41,726	€ 41,967	€ 41,129
**RWD-based analysis**						
	**Apixaban**	**VKA**	**Dabigatran**	**Rivaroxaban**		
Drug costs	€ 3,747 (10%)	€ 91 (<1%)	€ 3,232 (8%)	€ 3,532 (9%)		
Monitoring/ management costs	€ 1,059 (3%)	€ 2,017 (5%)	€ 1,034 (3%)	€ 1,048 (3%)		
Event costs	€ 16,922 (47%)	€ 19,522 (53%)	€ 18,793 (48%)	€ 17,865 (48%)		
Indirect costs	€ 13,974 (39%)	€ 15,535 (42%)	€ 16,224 (41%)	€ 14,869 (40%)		
*Total costs*	€ 35,703	€ 37,165	€ 39,284	€ 37,314		

Abbreviations: NMA, network meta-analysis; RWD, real-world data; VKA, vitamin K antagonist.

The deterministic results of the NMA-based and RWD-based analyses are summarized in Table 4. In NMA-based analysis, apixaban increases costs with €902 and QALYs with 0.262 over lifetime compared to VKA, resulting in an ICER of €3,506/QALY. In both analyses, all other ICERs were dominant, meaning that apixaban treatment is cost-saving while increasing patient’s health.

**Table 4:** Base-case results of the NMA-based and RWD-based analyses comparing apixaban to VKA and other NOACs.

Comparator	Incremental cost	Incremental QALY	Cost per QALY gained	Incremental LY	Cost per LY gained
**NMA-based analysis**					
VKA	€920	0.262	€3,506	0.269	€3,415
Dabigatran (110mg)	- €2,692	0.177	Dominant	0.207	Dominant
Dabigatran (150 mg)	- €819	0.131	Dominant	0.157	Dominant
Rivaroxaban	- €1,027	0.101	Dominant	0.126	Dominant
Edoxaban	- €197	0.065	Dominant	0.085	Dominant
**RWD-based analysis**					
VKA	- €1,358	0.330	Dominant	0.352	Dominant
Dabigatran	- €3,612	0.310	Dominant	0.383	Dominant
Rivaroxaban	- €1,625	0.124	Dominant	0.154	Dominant

Abbreviations: LY, life-years; NMA, network meta-analysis; QALY, quality adjusted life-years, RWD, real-world data; VKA, vitamin K antagonist.

### Sensitivity analyses

**Fig 2:**
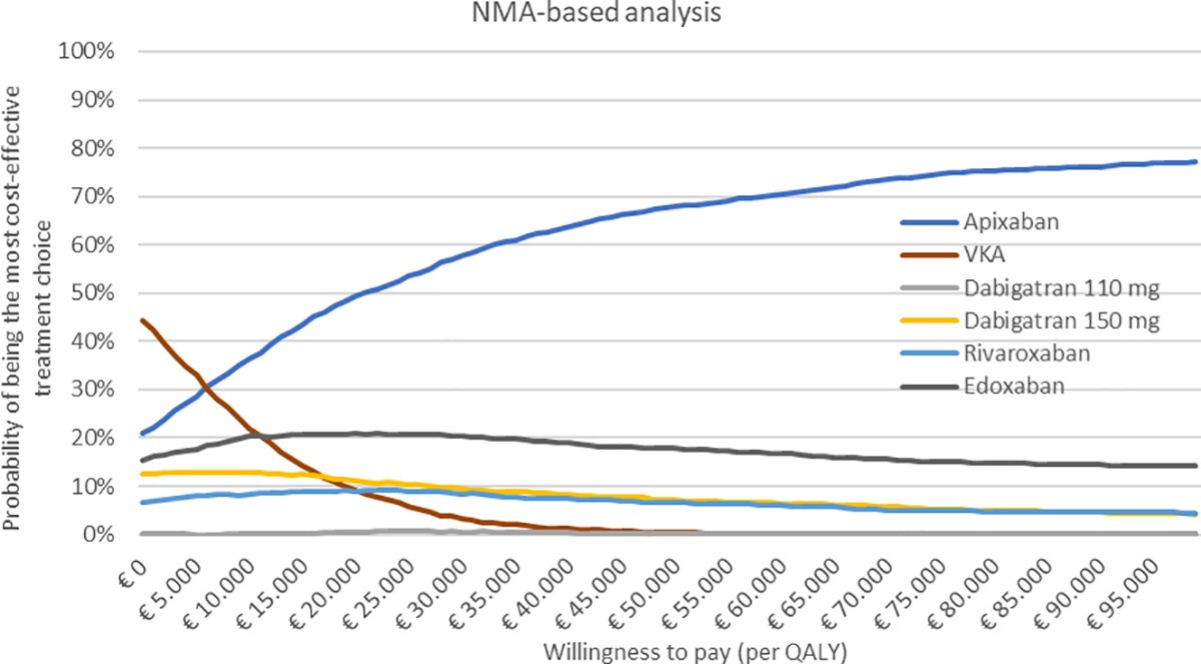
Probability of being the most cost-effective treatment choice per willingness-to-pay threshold for the NMA-based analysis. Abbreviations: NMA, network meta-analysis; QALY, quality adjusted life-years; VKA, vitamin K antagonist.

**Fig 3:**
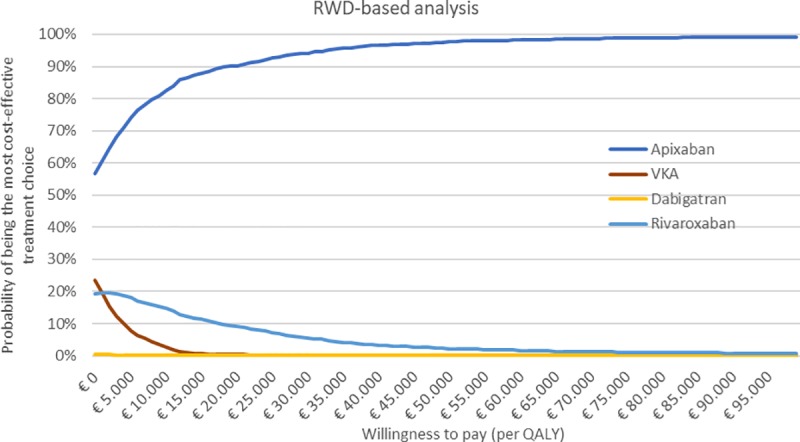
Probability of being the most cost-effective treatment choice per willingness-to-pay threshold for the RWD-based analysis. Abbreviations: QALY, quality adjusted life-years; RWD, real-world data; VKA, vitamin K antagonist.

The results of the PSA are plotted in cost-effectiveness planes, shown in S1 File. Figs 2 and 3 show the probability of being the most cost-effective treatment alternative per WTP threshold for the NMA-based and RWD-based analyses, respectively. In NMA-based analysis, apixaban is the most cost-effective treatment option at the €20,000/QALY WTP threshold with 50%. At the same threshold, the probabilities of VKA, dabigatran 110 mg, dabigatran 150 mg, rivaroxaban and edoxaban being the most cost-effective treatment option are: 8%, 1%, 11%, 9% and 21%, respectively. In RWD-based analysis, similar results were found: apixaban is the most cost-effective treatment with 90%, and apixaban was–compared to VKA, dabigatran and rivaroxaban respectively—cost-effective in 0%, 0% and 9% of the iterations. Nevertheless, apixaban was only significantly dominant compared to VKA in the RWD-based analysis, as in more than 95% of the PSA simulations apixaban was cost-saving and more effective compared to VKA. In all other PSAs the dominancy of apixaban was non-significant.

**Fig 4:**
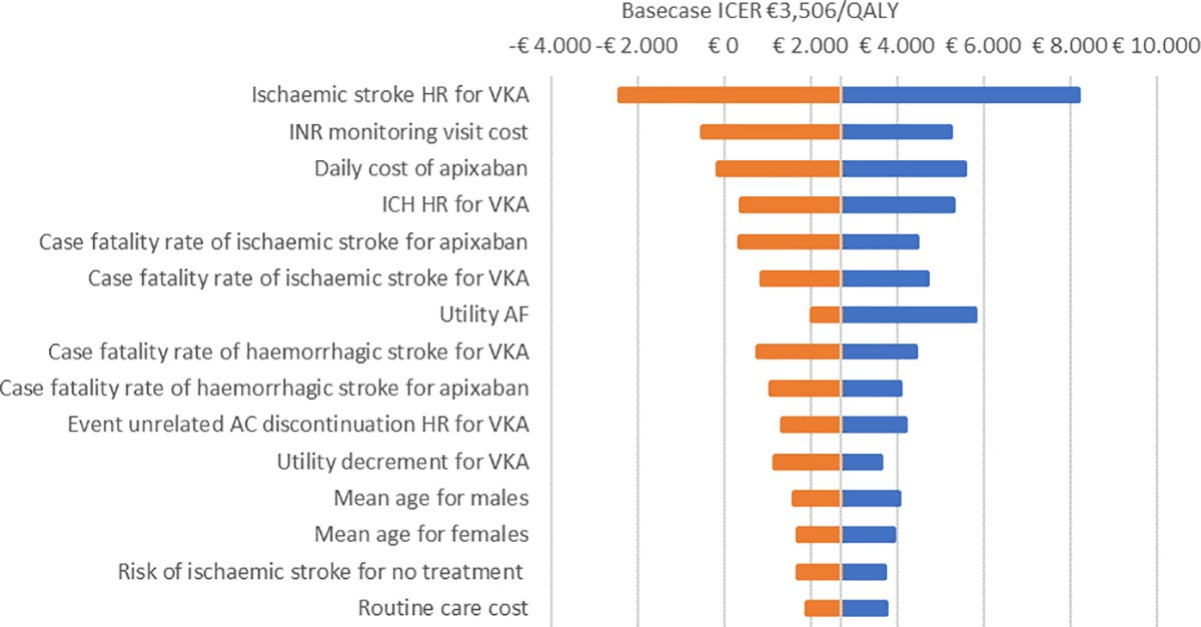
Univariate sensitivity analysis of NMA-based analysis comparing apixaban and VKA. **Figure depicts the influence of uncertainty in different input parameters on ICER.** Abbreviations: AF, atrial fibrillation; HR, hazard ratio, ICER, incremental cost-effectiveness ratio; ICH, intracranial haemorrhage; INR, international normalized ratio; QALY, quality adjusted life-years; SE, systemic embolism; VKA, vitamin K antagonist.

Additionally, we conducted a univariate sensitivity analysis. Fig 4 represents the tornado diagram for apixaban versus VKA (NMA-based analysis). Univariate sensitivity analyses comparing apixaban with other NOACs identified similar parameters to be the most influential on ICER. The parameters with the most influence are the ischaemic stroke and ICH hazard ratios for VKA compared to apixaban, INR monitoring costs, daily costs of apixaban and stroke (ischaemic or haemorrhagic) case fatalities.

### Scenario analyses

We conducted three additional scenarios to assess the influence of the healthcare payer’s perspective (scenario 1), equal drug costs among NOACs (scenario 2) and equal rates of event unrelated AC discontinuation among NOACs and VKAs (scenario 3). Scenario 1 showed that compared to societal perspective the incremental costs of apixaban versus VKA and edoxaban in the NMA-based analysis increase, resulting in an ICER of €5,787/QALY and €206/QALY.

In RWD-based analysis, apixaban is cost-effective compared to VKA (€292/QALY), and cost-saving (dominant) compared to dabigatran and rivaroxaban. In scenario 2, equal drug costs for NOACs resulted in an ICER of €2,884/QALY for apixaban versus edoxaban in the NMA-based analysis. All other ICERs are dominant.

In the third scenario of the NMA-based analysis, equal event unrelated AC discontinuation rates for NOACs and VKAs resulted in an ICER of €244,079/QALY for apixaban versus dabigatran 150 mg, which is not considered cost-effective at the WTP threshold of €20,000/QALY. In NMA-based analysis, apixaban appeared to be dominant over dabigatran 110 mg and rivaroxaban and cost-effective compared to VKA (€5,648/QALY) and edoxaban (€10,243/QALY). In RWD-based analysis apixaban was still dominant over VKA, dabigatran and rivaroxaban. Detailed results of the three scenarios are presented in Table 5.

**Table 5:** Results of the scenario analyses: NMA-based and RWD-based analyses calculated from healthcare payer's perspective (scenario 1), equal drugs costs for NOACs (scenario 2) and equal event unrelated AC discontinuation rates for NOACs and VKAs (scenario 3).

**Scenario 1: healthcare payer’s perspective**					
**Comparator**	**Incremental cost**	**Incremental QALY**	**Cost per QALY gained**	**Incremental LY**	**Cost per LY gained**
**NMA-based analysis**					
VKA	€1,518	0.262	€5,787	0.269	€5,636
Dabigatran (110mg)	- €1,122	0.177	Dominant	0.207	Dominant
Dabigatran (150 mg)	- €142	0.131	Dominant	0.157	Dominant
Rivaroxaban	- €277	0.101	Dominant	0.126	Dominant
Edoxaban	€13	0.065	€206	0.085	€157
**RWD-based analysis**					
VKA	€96	0.330	€292	0.352	€273
Dabigatran	- €1,331	0.310	Dominant	0.383	Dominant
Rivaroxaban	- €717	0.124	Dominant	0.154	Dominant
**Scenario 2: equal drug costs for NOACs**					
**Comparator**	**Incremental cost**	**Incremental QALY**	**Cost per QALY gained**	**Incremental LY**	**Cost per LY gained**
**NMA-based analysis**					
Dabigatran (110mg)	- €2,287	0.177	Dominant	0.207	Dominant
Dabigatran (150 mg)	- €411	0.131	Dominant	0.157	Dominant
Rivaroxaban	- €828	0.101	Dominant	0.126	Dominant
Edoxaban	€186	0.065	€2,884	0.085	€2,193
**RWD-based analysis**					
Dabigatran	- €3,244	0.330	Dominant	0.383	Dominant
Rivaroxaban	- €1,448	0.124	Dominant	0.154	Dominant
**Scenario 3: equal event unrelated AC discontinuation rate for NOACs and VKAs**					
**Comparator**	**Incremental cost**	**Incremental QALY**	**Cost per QALY gained**	**Incremental LY**	**Cost per LY gained**
**NMA-based analysis**					
VKA	€1,390	0.246	€5,648	0.249	€5,580
Dabigatran (110mg)	- €675	0.082	Dominant	0.103	Dominant
Dabigatran (150 mg)	€1,959	0.008	€244,079	0.022	€90,398
Rivaroxaban	- €100	0.056	Dominant	0.077	Dominant
Edoxaban	€385	0.038	€10,243	0.055	€6,951
**RWD-based analysis**					
VKA	- €1,241	0.326	Dominant	0.348	Dominant
Dabigatran	- €2,828	0.291	Dominant	0.373	Dominant
Rivaroxaban	- €1,215	0.100	Dominant	0.129	Dominant

Abbreviations: AC, anticoagulant; LY, life-years; NMA, network meta-analysis; QALY, quality adjusted life-year; RWD, real-world data; VKA, vitamin K antagonist.

## Discussion

To our knowledge, this is the first cost-effectiveness study comparing different NOACs and VKA with RCT data as well as RWD in patients with AF. The RWD-based analysis was specifically implemented to externally validate conclusions found in the NMA-based analysis based on RCT data. In the NMA-based analysis, apixaban appeared to be cost-effective compared to VKA and cost-saving compared to dabigatran 110 mg, dabigatran 150 mg, rivaroxaban and edoxaban. The RWD-based analysis showed that apixaban is cost-saving compared to all comparators. Apixaban was shown, in both analyses, to be the most cost-effective treatment option at a WTP threshold of €20,000/QALY (50% and 90%, respectively). In the scenario analysis apixaban appeared to be not cost-effective compared to dabigatran 150 mg, when using equal event-unrelated treatment discontinuation rates for each drug. In all other scenarios apixaban is cost-effective or cost-saving compared to VKA and other NOACs.

A previously published cost-effectiveness analysis, which was based on the same model as our analysis, was conducted in the UK setting [8]. They found that apixaban was cost-effective compared to dabigatran 110 mg, dabigatran 150 mg and rivaroxaban with ICERs of £4,497/QALY, £9,611/QALY and £5,305/QALY, respectively. In our analysis, we found apixaban to be dominant (cost-saving while increasing QALYs) over the other NOACs. This difference can be explained by the drug costs (apixaban is relatively lower priced in the Netherlands) as well as the difference in perspective, with UK consistently using the health-care perspective, i.e. that of the National Health Service (NHS). In our scenario analysis conducted from a healthcare payer’s perspective, the incremental costs did decrease compared to the societal perspective, supporting this hypothesis, although the ICERs remained dominant. Moreover, in our scenario analysis assuming equal AC costs apixaban was still dominant over all other NOACs except edoxaban in the NMA-based analysis, with an ICER of €2,884/QALY. This suggests that these differences in methodology are not solely the cause of the difference in ICERs.

In a follow-up publication [18], it was concluded that, apixaban was dominant over 30 mg and 60 mg edoxaban in the UK setting (with edoxaban not being included in the previous publication [8]). Two other studies in the UK calculated the cost-effectiveness based on alternative NMAs [36,37]. Both studies showed that, compared to the other NOACs, apixaban is the most cost-effective treatment option at a WTP threshold of £20,000/QALY.

A Dutch study that compared the cost-effectiveness of apixaban, rivaroxaban and dabigatran to VKA [38] found that from a healthcare payer's perspective apixaban was the most cost-effective option compared to VKA with an ICER of €14,626/QALY. The difference in outcome compared to our study can be explained by differences in input parameters (e.g. apixaban drug costs were higher compared to our analysis) and model structure. For example, they did not include haemorrhagic stroke costs and QALYs, which in our univariate analysis had the highest influence on the ICER. Also, they used the healthcare payer’s perspective and US tariffs for the quality of life, which might also contribute to different outcomes.

Event unrelated AC discontinuation rates differ among the anticoagulants [10]. To show the impact of these differences in event unrelated AC discontinuation rates on cost-effectiveness, we included a scenario assuming equal rates for all anticoagulants. This scenario shows to have a high influence on the ICER of apixaban compared to dabigatran 150 mg, which changed from dominant to €244,079/QALY. Apixaban remained cost-effective or cost-saving compared to the other NOACs and VKA. Firstly, dabigatran has the highest event unrelated AC discontinuation rate compared to apixaban (hazard ratio versus apixaban = 1.500). Secondly, dabigatran 150 mg has a better protective effect (hazard ratio versus apixaban = 0.790) compared to apixaban, while bleedings are a bit more frequent (hazard ratio versus apixaban ICH = 1.020 and other MB = 1.340). When assuming equal event unrelated AC discontinuation, the incremental QALYs of apixaban versus dabigatran 150 mg decreases from 0.131 in the base-case analysis to 0.008, resulting in a high ICER.

Notably, costs and QALYs are highly driven by the patients who discontinue AC therapy. Treatment discontinuation in patients with AF is very undesirable, because from that moment on they are not protected for stroke or other thromboembolic events. In practice, a certain percentage of the patients who discontinue their initial AC therapy, will be encouraged to switch to another AC. However, it is hard to get supportive data on the percentage of patients fully discontinuing AC therapy, as well as reliable data on what that exactly means for stroke and bleeding risks. By emphasizing the importance of AC therapy and discussing the reason for the AC discontinuation the patient can be encouraged to restart or switch AC therapy.

The major advantage of this study is that both an NMA and RWD were used for cost-effectiveness. For the RWD-based analysis we used the publication of Lip et al. that best met the inclusion criteria for the systematic literature search underlying the NMA [12]. Differences in estimated hazard ratios in NMA and RWD led to differences in results, although the overall outcomes of the RWD-based analysis support the conclusions of the NMA-based analysis.

Unfortunately, we were unable to include RWD on Dutch patients. Throughout the next years, a recently launched project “DUTCH-AF registry” aims to include 6,000 Dutch patients with AF to collect and assess data on effectiveness, adherence and bleeding risk of NOACs and VKAs [39]. It is interesting to have this data specific on the Dutch patients, since the patient characteristics, such as CHADS_2_/CHA_2_DS_2_-VASc scores and age, can obviously differ among populations. Obviously due to the fact that RWD studies include heterogeneous populations from different countries, differences in clinical outcomes can be expected, which potentially also leads to differences in economic outcomes. Therefore, the use of another RWD study might lead to different outcomes. Also, outcomes of NMAs can vary when making use of different combinations of clinical trials, differences in methodology and the differences in definitions of the outcomes [40], again potentially leading to other results than those based on our selected NMA and RWD.

Notably, we included low-dose dabigatran (110 mg) in our analysis. We included this dose since it was officially approved by European Medicines Agency [41], although it was approved with the condition that this low-dose should be reserved for patients older than 80 years and/or with a decreased renal function (eGFR<50 ml/min). Since this was not the population that was exclusively followed in the dabigatran (RE-LY) trial included in the NMA [15], the risks used in the model might not adequately represent the real-world situation, and possibly this leads to a undervaluation of the (cost-) effectiveness of dabigatran 110 mg.

## Conclusion

Based on RCT-data as well as RWD, we conclude that generally apixaban is cost-effective or even cost-saving (less costly and more effective) compared to VKA and other NOACs in the overall population of AF-patients. It should be mentioned that, especially compared to other NOACs, only marginal differences in costs and health effects were found and the use of other effectiveness and safety data sources or assumptions, considerations of specific patient populations, costs might lead to different outcomes.

## Supporting information

S1 Appendix Systematic literature search. (DOCX)

S2 Appendix Formulas. (DOCX)

S1 Table Patient baseline characteristics model inputs used in the NMA-based and RWD-based analyses. Abbreviations: CHADS2 score, congestive heart failure, hypertension, age ≥75 years, diabetes mellitus, prior stroke or transient ischemic attack or thromboembolism; CHA2DS2-VASc score, congestive heart failure, hypertension, age ≥75 years (2), diabetes mellitus, prior stroke or transient ischemic attack or thromboembolism (2) and vascular disease (peripheral arterial disease, previous MI, aortic atheroma).
(DOCX)

S2 Table Event rates for apixaban and VKA and dabigatran 110 mg, dabigatran 150 mg, rivaroxaban, and edoxaban and distributions of patients across different levels of ischaemic and haemorrhagic stroke severity. ^a^ The event rates of ischaemic stroke, ICH and other MB were adjusted by CHADS2-scores with the distribution presented in S1 Table. Abbreviations: AC, anticoagulant; CHADS2, congestive heart failure, hypertension, age ≥75 years, diabetes mellitus, prior stroke or transient ischemic attack or thromboembolism; CI, confidence interval; CRNMB, clinically relevant non-major bleeding; CV, cardiovascular; GI, gastro-intestinal; HR, hazard ratio; ICH, intracranial haemorrhage; MB, major bleed; MI, myocardial infarction; NOAC, non-vitamin K antagonist oral anticoagulant; PY, patient-years; SE, systemic embolism; VKA, vitamin K antagonist.
(DOCX)

S3 Table Input parameters for the RWD-based analysis obtained from real-world study comparing apixaban with VKA and other NOACs by Lip et al. [3]. Abbreviations: CI, confidence interval; HR, hazard ratio; ICH, intracranial haemorrhage; MB, major bleeding, PY, patient-years; SE, systemic embolism; VKA, vitamin K antagonist. ^a^ *Hazard ratio apixaban versus comparator* = (1/*HR comparator versus apixaban*).
(DOCX)

S4 Table Background mortality, case fatality and mortality risk adjustment factors per event. ^a^ Lambda and Gamma are the natural logarithms of the slope of the survival hazard and age, respectively, which can be used to calculate the predicted survival at any time per age group (0–75 or >75 years) and gender. ^b^ Assumed to be equal to mortality risk adjustment factor of AF, since these events we assumed to have no additional effect on mortality risk in the period after the event. Abbreviations: AF, atrial fibrillation; CI, confidence interval; CRNMB, clinically relevant non-major bleeding; HR, hazard ratio; ICH, intracranial haemorrhage; MB, major bleeding; MI, myocardial infarction; SE, systemic embolism.
(DOCX)

S5 Table Event rates per 100 patient-years for no treatment after event unrelated treatment discontinuation. ^a^ intracranial haemorrhage including haemorrhagic stroke. Abbreviations: CI, confidence interval; CRNMB, clinically relevant non-major bleeding; CV, cardiovascular; ICH, intracranial haemorrhage; MB, major bleeding; MI, myocardial infarction; PY, patient-years; SE, systemic embolism.
(DOCX)

S1 File Probabilistic sensitivity analysis results. (DOCX)
