# Correction: Cost-effectiveness of apixaban compared to other anticoagulants in patients with atrial fibrillation in the real-world and trial settings

**DOI:** 10.1371/journal.pone.0266625

**Published:** 2022-03-31

**Authors:** Lisa A. de Jong, Jessie Groeneveld, Jelena Stevanovic, Harrie Rila, Robert G. Tieleman, Menno V. Huisman, Maarten J. Postma, Marinus van Hulst

Following the publication of this article [1] the authors received additional information that a correction [2] was published on an article [3] which the authors used for the real-world data (RWD) analysis. The authors were notified that a proportion of Medicare patients from the CMS database were unintentionally omitted from the original analysis [3]. The published corrected study [2] now incorporates the complete dataset.

As a result, the authors have re-analyzed the RWD-based analysis based on the corrected dataset reported in [2]. This has resulted in numerical changes to the outcomes of RWD analyses, which are reflected in the updated versions of Fig 3, Tables 3, 4 and 5, and Supporting Information files provided with this notice.

**Fig 3:**
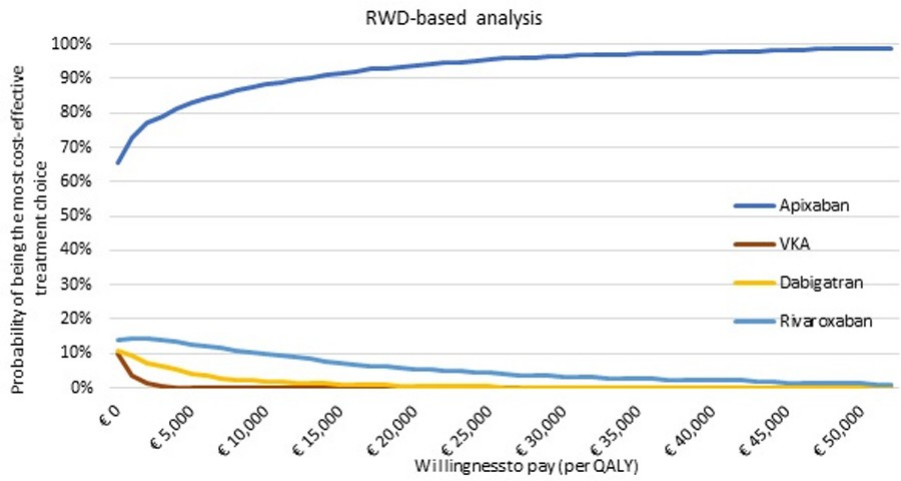
Probability of being the most cost-effective treatment choice per willingness-to-pay threshold for the RWD-based analysis. Abbreviations: QALY, quality adjusted life-years; RWD, real-world data; VKA, vitamin K antagonist. threshold for the RWD-based analysis.

**Table 3:** Base-case costs outcomes of the NMA-based and RWD-based analyses presented as costs per patient over a lifetime horizon.

**NMA-based analysis**						
	**Apixaban**	**VKA**	**Dabigatran 110 mg**	**Dabigatran 150 mg**	**Rivaroxaban**	**Edoxaban**
Drug costs	€ 3,925 (10%)	€ 95 (<1%)	€ 3,426 (8%)	€ 3,323 (8%)	€ 3,683 (9%)	€ 4,020 (10%)
Monitoring/ management costs	€ 1,181 (3%)	€ 2,192 (5%)	€ 1,148 (3%)	€ 1,179 (3%)	€ 1,174 (3%)	€ 1,176 (3%)
Event costs	€ 18,573 (45%)	€ 19,872 (49%)	€ 20,227 (46%)	€ 19,320 (46%)	€ 19,100 (46%)	€ 18,470 (45%)
Indirect costs	€ 17,289 (42%)	€ 18,005 (45%)	€ 18,811 (43%)	€ 17,905 (43%)	€ 18,010 (43%)	€ 17,463 (42%)
*Total costs*	€ 40,968	€ 40,163	€ 43,612	€ 41,726	€ 41,967	€ 41,129
**RWD-based analysis**						
	**Apixaban**	**VKA**	**Dabigatran**	**Rivaroxaban**		
Drug costs	€ 3,661 (12%)	€ 89 (<1%)	€ 3,171 (9%)	€ 3,471 (10%)		
Monitoring/ management costs	€ 1,000 (3%)	€ 1,940 (6%)	€ 984 (3%)	€ 990 (3%)		
Event costs	€ 15,208 (48%)	€ 17,339 (53%)	€ 16,383 (48%)	€ 16,118 (48%)		
Indirect costs	€ 11,878 (37%)	€ 13,051 (40%)	€ 13,307 (39%)	€ 12,740 (38%)		
*Total costs*	€ 31,747	€ 32,419	€ 33,845	€ 33,714		

Abbreviations: NMA, network meta-analysis; RWD, real-world data; VKA, vitamin K antagonist.

**Table 4:** Base-case results of the NMA-based and RWD-based analyses comparing apixaban to VKA and other NOACs.

Comparator	Incremental cost	Incremental QALY	Cost per QALY gained	Incremental LY	Cost per LY gained
**NMA-based analysis**					
VKA	€920	0.262	€3,506	0.269	€3,415
Dabigatran (110mg)	- €2,692	0.177	Dominant	0.207	Dominant
Dabigatran (150 mg)	- €819	0.131	Dominant	0.157	Dominant
Rivaroxaban	- €1,027	0.101	Dominant	0.126	Dominant
Edoxaban	- €197	0.065	Dominant	0.085	Dominant
**RWD-based analysis**					
VKA	- €672	0.285	Dominant	0.299	Dominant
Dabigatran	- €2,098	0.216	Dominant	0.266	Dominant
Rivaroxaban	- €1,571	0.113	Dominant	0.140	Dominant

Abbreviations: LY, life-years; NMA, network meta-analysis; QALY, quality adjusted life-years, RWD, real-world data; VKA, vitamin K antagonist.

**Table 5:** Results of the scenario analyses: NMA-based and RWD-based analyses calculated from healthcare payer’s perspective (scenario 1), equal drugs costs for NOACs (scenario 2) and equal event unrelated AC discontinuation rates for NOACs and VKAs (scenario 3).

**Scenario 1: healthcare payer’s perspective**					
**Comparator**	**Incremental cost**	**Incremental QALY**	**Cost per QALY gained**	**Incremental LY**	**Cost per LY gained**
**NMA-based analysis**					
VKA	€1,518	0.262	€5,787	0.269	€5,636
Dabigatran (110mg)	- €1,122	0.177	Dominant	0.207	Dominant
Dabigatran (150 mg)	- €142	0.131	Dominant	0.157	Dominant
Rivaroxaban	- €277	0.101	Dominant	0.126	Dominant
Edoxaban	€13	0.065	€206	0.085	€157
**RWD-based analysis**					
VKA	€498	0.285	€1,750	0.299	€1,668
Dabigatran	- €669	0.216	Dominant	0.266	Dominant
Rivaroxaban	- €943	0.137	Dominant	0.170	Dominant
**Scenario 2: equal drug costs for NOACs**					
**Comparator**	**Incremental cost**	**Incremental QALY**	**Cost per QALY gained**	**Incremental LY**	**Cost per LY gained**
**NMA-based analysis**					
Dabigatran (110mg)	- €2,287	0.177	Dominant	0.207	Dominant
Dabigatran (150 mg)	- €411	0.131	Dominant	0.157	Dominant
Rivaroxaban	- €828	0.101	Dominant	0.126	Dominant
Edoxaban	€186	0.065	€2,884	0.085	€2,193
**RWD-based analysis**					
Dabigatran	- €1,767	0.216	Dominant	0.266	Dominant
Rivaroxaban	- €160	0.137	Dominant	0.170	Dominant
**Scenario 3: equal event unrelated AC discontinuation rate for NOACs and VKAs**					
**Comparator**	**Incremental cost**	**Incremental QALY**	**Cost per QALY gained**	**Incremental LY**	**Cost per LY gained**
**NMA-based analysis**					
VKA	€1,390	0.246	€5,648	0.249	€5,580
Dabigatran (110mg)	- €675	0.082	Dominant	0.103	Dominant
Dabigatran (150 mg)	€1,959	0.008	€244,079	0.022	€90,398
Rivaroxaban	- €100	0.056	Dominant	0.077	Dominant
Edoxaban	€385	0.038	€10,243	0.055	€6,951
**RWD-based analysis**					
VKA	- €445	0.279	Dominant	0.291	Dominant
Dabigatran	- €1,160	0.173	Dominant	0.224	Dominant
Rivaroxaban	- €1,666	0.115	Dominant	0.147	Dominant

Abbreviations: AC, anticoagulant; LY, life-years; NMA, network meta-analysis; QALY, quality adjusted life-year; RWD, real-world data; VKA, vitamin K antagonist.

Statements in [1] that were affected by the re-analysis are listed and corrected in the table below titled, “Table 6. Text Corrections”. In this table, reference 12a is used to designate Lip et al. (2020) (listed as reference [2] in this Correction).

**Table 6:** Text Corrections.

Location	Original text	Corrected text
Methods, second paragraph, fifth sentence	Following the pre-defined eligibility criteria, the real-world study of Lip et al. [12] was considered the most appropriate for use in the RWD-based analysis.	Following the pre-defined eligibility criteria, the real-world study of Lip et al. [12,12a] was considered the most appropriate for use in the RWD-based analysis.
Methods, Patient characteristics section, fifth sentence	The patients were on average 74.3 years old, 54.1% were male and the average CHA_2_DS_2_-VASc score was 3.7.	The patients were on average 76.1 years old, 51.4% were male and the average CHA_2_DS_2_-VASc score was 3.9.
Methods, Transition probabilities section, Event rates subsection, second paragraph, second sentence	Based on the real-world study by Lip et al. [12] we included RWD-based event rates of apixaban and VKA and hazard ratios of dabigatran and rivaroxaban for ischaemic stroke, ICH, other MB and SE, and distributions of haemorrhagic stroke among ICH and GI bleeding among other MB.	Based on the real-world study by Lip et al. [12,12a]] we included RWD-based event rates of apixaban and VKA and hazard ratios of dabigatran and rivaroxaban for ischaemic stroke, ICH, other MB and SE, and distributions of haemorrhagic stroke among ICH and GI bleeding among other MB.
Results, Deterministic results section, first paragraph, first sentence	Table 3 summarizes the costs outcomes per category. Event costs are the largest contributor to the total costs (45–49% and 47–53% in the NMA-based and RWD-based analyses, respectively).	Table 3 summarizes the costs outcomes per category. Event costs are the largest contributor to the total costs (45–49% and 48–53% in the NMA-based and RWD-based analyses, respectively).
Results, Deterministic results section, first paragraph, second sentence	Indirect costs also have high impact on the total costs: in both analyses 39–45% of the total costs are related to indirect costs.	Indirect costs also have high impact on the total costs: in both analyses 37–45% of the total costs are related to indirect costs.
Results, Deterministic results section, first paragraph, third sentence	In VKA treated patients, the impact of drug costs is negligible compared to NOACs (<1%% vs. 8–10% of total costs).	In VKA treated patients, the impact of drug costs is negligible compared to NOACs (<1% vs. 8–12% of total costs).
Results, Sensitivity analyses section, first paragraph, sentences 5 and 6	In RWD-based analysis, similar results were found: apixaban is the most cost-effective treatment with 90%, and apixaban was–compared to VKA, dabigatran and rivaroxaban respectively—cost-effective in 0%, 0% and 9% of the iterations. Nevertheless, apixaban was only significantly dominant compared to VKA in the RWD-based analysis, as in more than 95% of the PSA simulations apixaban was cost-saving and more effective compared to VKA.	In RWD-based analysis, similar results were found: apixaban is the most cost-effective treatment with 94%, and apixaban was–compared to VKA, dabigatran and rivaroxaban respectively—cost-effective in 0%, 0% and 5% of the iterations. Nevertheless, apixaban was only significantly dominant compared to VKA in the RWD-based analysis, as in more than 89% of the PSA simulations apixaban was cost-saving and more effective compared to VKA.
Results, Scenario analyses section, second paragraph, first sentence	In RWD-based analysis, apixaban is cost-effective compared to VKA (€292/QALY), and cost-saving (dominant) compared to dabigatran and rivaroxaban.	In RWD-based analysis, apixaban is cost-effective compared to VKA (€1,750/QALY), and cost-saving (dominant) compared to dabigatran and rivaroxaban.
Discussion, first paragraph, fifth sentence	Apixaban was shown, in both analyses, to be the most cost-effective treatment option at a WTP threshold of €20,000/QALY (50% and 90%, respectively).	Apixaban was shown, in both analyses, to be the most cost-effective treatment option at a WTP threshold of €20,000/QALY (50% and 94%, respectively).
Discussion, seventh paragraph, first sentence	The major advantage of this study is that both an NMA and RWD were used for cost-effectiveness. For the RWD-based analysis we used the publication of Lip et al. that best met the inclusion criteria for the systematic literature search underlying the NMA [12].	The major advantage of this study is that both an NMA and RWD were used for cost-effectiveness. For the RWD-based analysis we used the publication of Lip et al. that best met the inclusion criteria for the systematic literature search underlying the NMA [12,12a].

The S3 Table legend in [1] cited reference 3 for Lip et al. (2018), which aligns with the reference number in S1 Appendix rather than the reference number in the article’s main reference list. The legend has been updated, below, to cite publication years for the Lip et al. article (2018) and correction (2020) [2].

An Editorial Board member has reviewed the updates to the RWD-based analysis and determined that the conclusions of article [1] are upheld.

## Supporting information

The following are corrected versions of the Supporting Information files reported in [1].

S1 Table Patient baseline characteristics model inputs used in the NMA-based and RWD-based analyses. (DOCX)

S2 Table Event rates for apixaban and VKA and dabigatran 110 mg, dabigatran 150 mg, rivaroxaban, and edoxaban and distributions of patients across different levels of ischaemic and haemorrhagic stroke severity. (DOCX)

S3 Table Input parameters for the RWD-based analysis obtained from real-world study comparing apixaban with VKA and other NOACs by Lip et al. (2018, 2020). (DOCX)

S4 Table Background mortality, case fatality and mortality risk adjustment factors per event. (DOCX)

S5 Table Event rates per 100 patient-years for no treatment after event unrelated treatment discontinuation. (DOCX)

S1 File Probabilistic sensitivity analysis results. (DOCX)
