# Supplementary material for: Cost-effectiveness of apixaban compared to other anticoagulants in patients with atrial fibrillation in the real-world and trial settings
Source: PLoS One. 2019 Sep 17;14(9):e0222658. doi: 10.1371/journal.pone.0222658 (PMC6748426; doi:10.1371/journal.pone.0222658)
Supplement: S1 Appendix — (DOCX) [file pone.0222658.s001.docx]

S1 Appendix - Systematic literature search

## **Method of systematic literature search**

The literature search was performed in the PubMed database to identify studies assessing the real-world effectiveness and safety of NOACs. We used combinations of the following MESH terms: “real world” OR “observational” OR “registry” AND “atrial fibrillation” AND “apixaban”. Articles had to be published in English within the time frame of January 1 2012 to December 1 2018 and had to be peer reviewed. Remaining articles were checked for other relevant publications.

All records were screened based on title and abstract and selected based on the study design, included comparators and outcomes. Real-world observational studies (e.g. prospective and retrospective cohort studies and database or registry studies) as well as meta-analyses combining different real-world observational studies were screened for inclusion. The study should report all the primary endpoints of the ARISTOTLE trial (ischaemic stroke, SE, MB, ICH, haemorrhagic stroke) [1]. Since the model discriminates between ischaemic stroke and SE, these outcomes needed to be reported separately (not as composite). Outcomes had to be reported as HRs comparing apixaban to other NOACs (and VKA). The study should at least compare apixaban, dabigatran and rivaroxaban, with or without including VKA. Edoxaban received European Medicines Agency approval in 2015, and is therefore often not yet included in real-world studies [2]. The indirect comparative RWD studies had to make use of propensity score adjustment, to account for differences in characteristics (e.g. CHA_2_DS_2_-VASc score) between groups. To prevent double counting, publications reporting data for the same patients were evaluated and only the publication with the most representative population was included.

The study considered most appropriate by satisfying all above mentioned eligibility criteria for the secondary analysis was implemented in the model. All missing non-primary outcomes, such as treatment discontinuation or MI, were assumed to remain similar to the NMA-based analysis. Outcomes of the deterministic and sensitivity analysis were presented, and used to show the cost-effectiveness of different NOACs in real-world setting.

## **Results of systematic literature search**

Literature searches in PubMed identified a total of 260 records and 3 articles were identified through other sources (see PRISMA flow diagram below). After removal of duplicates, 226 records remained. During title and abstract screening 180 records were excluded, of which most studies did not meet the interest of our study (e.g. no RWD, irrelevant study design). After full-text screening (46 articles), one study was considered most appropriate for use in the secondary analysis [3]. Forty-four of the full-text screened articles did not meet inclusion criteria: 15 studies did not include all relevant comparators and 25 studies reported not all outcomes of interest. One study compared the different NOACs with odds ratios [2] and three studies only reported HRs of all primary outcomes of NOACs compared to VKA, while not comparing the NOACs to each other [4–6]. Two studies did meet all the inclusion criteria but had possible overlap in included patients [3,7]. Of which the article of Lip et al. [3] was considered the most appropriate for use in the secondary analysis, since they included the biggest population using different databases, among which the same database as used by Deitelzweig et al. [7]. In total, Lip analysed 321,182 AF-patients on warfarin, apixaban, dabigatran and rivaroxaban. The patients were on average 74 years old and 54% was men. A proportion of the NOAC treated patients received reduced dose (22.5%, 15.4% and 27.1% for apixaban, dabigatran and rivaroxaban, respectively). Since drug prices do not differ among the different doses we did not have to adjust any costs [8]. In RWD-based analysis, we considered the patient characteristics, event rates and HRs as provided in Lip et al. (S3 Table).

**PRISMA flow diagram for systematic literature review used to identify real-world studies eligible for use in a secondary analysis based on RWD.** NOACs, non-vitamin K antagonist oral anticoagulants.

# **References**

1. Granger CB, Alexander JH, McMurray JJV, Lopes RD, Hylek EM, Hanna M, et al. Apixaban versus Warfarin in Patients with Atrial Fibrillation. N Engl J Med. 2011;365(11):981–92.

2. Committee for Medicinal Products for Human Use (CHMP). Committee for Medicinal Products for Human Use (CHMP) Assessment report. 2015.

3. Lip GY, Keshishian A, Li X, Hamilton M, Masseria C, Gupta K, et al. Effectiveness and Safety of Oral Anticoagulants Among Nonvalvular Atrial Fibrillation Patients. Stroke. 2018;49(0):00.

4. Amin A, Keshishian A, Trocio J, Dina O, Le H, Rosenblatt L, et al. Risk of stroke/systemic embolism, major bleeding and associated costs in non-valvular atrial fibrillation patients who initiated apixaban, dabigatran or rivaroxaban compared with warfarin in the United States Medicare population. Curr Med Res Opin. 2017;33(9):1595–604.

5. Hohnloser SH, Basic E, Hohmann C, Nabauer M. Effectiveness and Safety of Non-Vitamin K Oral Anticoagulants in Comparison to Phenprocoumon: Data from 61,000 Patients with Atrial Fibrillation. Thromb Haemost. 2018;118(3):526–38.

6. Noseworthy PA, Yao X, Abraham NS, Sangaralingham LR, McBane RD, Shah ND. Direct Comparison of Dabigatran, Rivaroxaban, and Apixaban for Effectiveness and Safety in Nonvalvular Atrial Fibrillation. Chest. 2016;150(6):1302–12.

7. Deitelzweig S, Luo X, Gupta K, Trocio J, Mardekian J, Curtice T, et al. Comparison of effectiveness and safety of treatment with apixaban vs. other oral anticoagulants among elderly nonvalvular atrial fibrillation patients. Curr Med Res Opin. 2017;33(10):1745–54.

8. Z-index. The Pharmacy Purchase Price - apixaban. July [Internet]. 2018; Available from: https://www.z-index.nl/

9. Korenstra J, Petra E, Wijtvliet J, Veeger NJGM, Geluk CA, Bartels GL, et al. Effectiveness and safety of dabigatran versus acenocoumarol in “real-world” patients with atrial fibrillation. Europace. 2016;18:1319–27.

10. Lip GYH, Mitchell SA, Liu X, Liu LZ, Phatak H, Kachroo S, et al. Relative efficacy and safety of non-Vitamin K oral anticoagulants for non-valvular atrial fibrillation: Network meta-analysis comparing apixaban, dabigatran, rivaroxaban and edoxaban in three patient subgroups. Int J Cardiol. 2016;204:88–94.

11. Connolly SJ, Eikelboom J, Joyner C, Diener H-C, Hart R, Golitsyn S, et al. Apixaban in Patients with Atrial Fibrillation. N Engl J Med. 2011;364(9):806–17.

12. Connolly SJ, Ezekowitz MD, Yusuf S, Eikelboom J, Oldgren J, Parekh A, et al. Dabigatran versus Warfarin in Patients with Atrial Fibrillation. N Engl J Med. 2009;361(12):1139–51.

13. Giugliano RP, Ruff CT, Braunwald E, Murphy SA, Wiviott SD, Halperin JL, et al. Edoxaban versus Warfarin in Patients with Atrial Fibrillation. N Engl J Med. 2013;369(22):2093–104.

14. Patel MR, Mahaffey KW, Garg J, Pan G, Singer DE, Hacke W, et al. Rivaroxaban versus Warfarin in Nonvalvular Atrial Fibrillation. N Engl J Med. 2011;365(10):883–91.

15. Statistics Netherlands (CBS). Life expectancy; sex, age (per year and period of five years) [Internet]. [cited 2018 Jun 14]. Available from: https://statline.cbs.nl/StatWeb/

16. Friberg L, Hammar N, Pettersson H, Rosenqvist M. Increased mortality in paroxysmal atrial fibrillation: report from the Stockholm Cohort-Study of Atrial Fibrillation (SCAF). Eur Heart J. 2007;28(19):2346–53.

17. Brønnum-Hansen H, Davidsen M, Thorvaldsen P, Danish MONICA Study Group. Long-term survival and causes of death after stroke. Stroke. 2001;32(9):2131–6.

18. Henriksson KM, Farahmand B, Johansson S, Asberg S, Terént A, Edvardsson N. Survival after stroke--the impact of CHADS2 score and atrial fibrillation. Int J Cardiol. 2010;141(1):18–23.

19. Huybrechts KF, Caro JJ, Xenakis JJ, Vemmos KN. The prognostic value of the modified Rankin Scale score for long-term survival after first-ever stroke. Results from the Athens Stroke Registry. Cerebrovasc Dis. 2008;26(4):381–7.

20. Brønnum-Hansen H, Jørgensen T, Davidsen M, Madsen M, Osler M, Gerdes LU, et al. Survival and cause of death after myocardial infarction: the Danish MONICA study. J Clin Epidemiol. 2001;54(12):1244–50.

21. Mandema J. Meta analysis of placebo, ASA and warfarin controlled studies in AF. Data file. 2011;

22. Hylek EM, Go AS, Chang Y, Jensvold NG, Henault LE, Selby J V., et al. Effect of Intensity of Oral Anticoagulation on Stroke Severity and Mortality in Atrial Fibrillation. N Engl J Med. 2003;349(11):1019–26.
