# Supplementary material for: Cost-effectiveness of apixaban compared to other anticoagulants in patients with atrial fibrillation in the real-world and trial settings
Source: PLoS One. 2019 Sep 17;14(9):e0222658. doi: 10.1371/journal.pone.0222658 (PMC6748426; doi:10.1371/journal.pone.0222658)
Supplement: S2 Appendix — (DOCX) [file pone.0222658.s002.docx]

S2 Appendix - Formulas

Formulas used to calculate per cycle event rates for apixaban and VKA:

$$Event rate per day=0.01*\frac{event rate per 100 PY}{365.25}$$

$$Event rate per cycle=1-e^{-(event rate per day*42)}$$

Formula used to calculate per cycle event rates for other NOACs:

$${Event rate per cycle}_{other NOAC}={HR}_{other NOAC}*{event rate per cycle}_{apixaban}$$
