# Supplementary material for: Cost-effectiveness of apixaban compared to other anticoagulants in patients with atrial fibrillation in the real-world and trial settings
Source: PLoS One. 2019 Sep 17;14(9):e0222658. doi: 10.1371/journal.pone.0222658 (PMC6748426; doi:10.1371/journal.pone.0222658)
Supplement: S1 Table — Abbreviations: CHADS2 score, congestive heart failure, hypertension, age ≥75 years, diabetes mellitus, prior stroke or transient ischemic attack or thromboembolism; CHA2DS2-VASc score, congestive heart failure, hypertension, age ≥75 years (2), diabetes mellitus, prior stroke or transient ischemic attack or thromboembolism (2) and vascular disease (peripheral arterial disease, previous MI, aortic atheroma). (DOCX) [file pone.0222658.s003.docx]

S1 Table

**Patient baseline characteristics model inputs used in the NMA-based and RWD-based analyses.**

| **Characteristic** | **Value (mean)** | **Reference** |
| --- | --- | --- |
| **NMA-based analysis** | | |
| Age |  |  |
| Male (years) | 71.5 | [9] |
| Female (years) | 71.5 | [9] |
| Gender (male, %) | 64.7 | [9] |
| CHADS_2_ distribution |  |  |
| 0-1 (%) | 50.7 | [9] |
| 2 (%) | 29.1 | [9] |
| >3 (%) | 20.2 | [9] |
| Average CHADS_2_ score | 1.7 | [9] |
| Average CHA_2_DS_2_-VASc score | 3.1 | [9] |
| **RWD-based analysis** | | |
| Age |  |  |
| Male (years) | 74.3 | [3] |
| Female (years) | 74.3 | [3] |
| Gender (male, %) | 54.1 | [3] |
| CHA_2_DS_2_-VASc score distribution |  |  |
| 0-1 (%) | 9.8 | [3] |
| 2 (%) | 14.5 | [3] |
| >3 (%) | 75.7 | [3] |
| Average CHA_2_DS_2_-VASc score | 3.7 | [3] |

Abbreviations: CHADS_2_ score, congestive heart failure, hypertension, age ≥75 years, diabetes mellitus, prior stroke or transient ischemic attack or thromboembolism; CHA_2_DS_2_-VASc score, congestive heart failure, hypertension, age ≥75 years (2), diabetes mellitus, prior stroke or transient ischemic attack or thromboembolism (2) and vascular disease (peripheral arterial disease, previous MI, aortic atheroma)
