# Supplementary material for: Cost-effectiveness of apixaban compared to other anticoagulants in patients with atrial fibrillation in the real-world and trial settings
Source: PLoS One. 2019 Sep 17;14(9):e0222658. doi: 10.1371/journal.pone.0222658 (PMC6748426; doi:10.1371/journal.pone.0222658)
Supplement: S2 Table — a The event rates of ischaemic stroke, ICH and other MB were adjusted by CHADS2-scores with the distribution presented in S1 Table. Abbreviations: AC, anticoagulant; CHADS2, congestive heart failure, hypertension, age ≥75 years, diabetes mellitus, prior stroke or transient ischemic attack or thromboembolism; CI, confidence interval; CRNMB, clinically relevant non-major bleeding; CV, cardiovascular; GI, gastro-intestinal; HR, hazard ratio; ICH, intracranial haemorrhage; MB, major bleed; MI, myocardial infarction; NOAC, non-vitamin K antagonist oral anticoagulant; PY, patient-years; SE, systemic embolism; VKA, vitamin K antagonist. (DOCX) [file pone.0222658.s004.docx]

S2 Table

**Event rates for apixaban and VKA and dabigatran 110 mg, dabigatran 150 mg, rivaroxaban, and edoxaban and distributions of patients across different levels of ischaemic and haemorrhagic stroke severity.**

|  | **Event rate per 100 PY** | | **HR other NOAC vs apixaban (95% CI)** | | | | **Source** |
| --- | --- | --- | --- | --- | --- | --- | --- |
|  | **Apixaban** | **VKA** | **Dabigatran (110 mg)** | **Dabigatran (150 mg)** | **Rivaroxaban** | **Edoxaban** |  |
| Ischaemic stroke ^a^ |  |  |  |  |  |  |  |
| CHADS_2_ 0-1 | 0.521 (0.355-0.718) | 0.458 (0.434-0.686) | 1.170 (0.850-1.620) | 0.790 (0.560-1.100) | 1.030 (0.770-1.380) | 1.040 (0.780-1.390) | [1,10] |
| CHADS_2_ 2 | 0.950 (0.719-1.212) | 0.950 (0.791-1.250) |  |  |  |  |  |
| CHADS_2_ ≥ 3 | 1.534 (1.205-1.902) | 1.944 (1.278-2.019) |  |  |  |  |  |
| ICH ^a^ |  |  |  |  |  |  |  |
| CHADS_2_ 0-1 | 0.252 (0.144-0.390) | 0.559 (0.444-0.852) | 0.730 (0.430-1.260) | 1.020 (0.620-1.680) | 1.730 (1.080-2.770) | 1.110 (0.710-1.730) | [1,10] |
| CHADS_2_ 2 | 0.348 (0.199-0.538) | 0.582 (0.612-1.176) |  |  |  |  |  |
| CHADS_2_ ≥ 3 | 0.328 (0.187-0.507) | 1.127 (0.577-1.109) |  |  |  |  |  |
| Other MB ^a^ |  |  |  |  |  |  |  |
| CHADS_2_ 0-1 | 1.103 (0.630-1.706) | 1.714 (0.980-2.502) | 1.180 (0.940-1.470) | 1.340 (1.080-1.670) | 1.410 (1.130-1.750) | 1.130 (0.910-1.390) | [1,10] |
| CHADS_2_ 2 | 1.898 (1.085-2.935) | 2.360 (1.349-3.446) |  |  |  |  |  |
| CHADS_2_ ≥ 3 | 2.500 (1.429-3.866) | 2.821 (1.612-4.119) |  |  |  |  |  |
| CRNMB | 2.083 (1.191-3.221) | 2.995 (2.625-3.562) | 1.000 (0.900-1.100) | 1.000 (0.900-1.100) | 1.520 (1.280-10) | 1.250 (1.060-1.480) | [1,10] |
| MI | 0.530 (0.303-0.820) | 0.610 (0.461-0.811) | 1.470 (0.980-2.230) | 1.460 (0.960-2.200) | 1.060 (0.730-1.520) | 1.070 (0.740-1.550) | [1,10] |
| SE | 0.090 (0.051-0.139) | 0.100 (0.057-0.155) | 0.780 (0.300-2.050) | 0.720 (0.270-1.920) | 0.850 (0.350-2.060) | 0.740 (0.290-1.920) | [1,10] |
| Other CV hospitalization | 10.460 (5.979-16.174) | 10.460 (5.979-16.174) | 1.000 (0.900-1.100) | 1.000 (0.900-1.100) | 1.000 (0.900-1.100) | 1.000 (0.900-1.100) | [11] |
| Event unrelated AC discontinuation | 13.177 (7.532-20.375) | 14.405 (8.234-22.274) | 1.450 (1.310-1.610) | 1.500 (1.360-1.670) | 1.180 (1.080-1.290) | 1.100 (1.010-1.190) | [1,10] |
|  | **Distribution of severity or origin of event (% of patients)** | | | | | | |
|  | **Apixaban** | **VKA** | **Dabigatran (110 mg)** | **Dabigatran (150 mg)** | **Rivaroxaban** | **Edoxaban** | **Source** |
| Ischaemic stroke |  | | | | | | |
| Mild | 53 (44-62) | 45 (36-54) | 35 (28-42) | 35 (28-42) | 49 (42-56) | 47 (40-54) | [1,10] |
| Moderate | 21 (14-29) | 30 (22-39) | 28 (22-35) | 22 (16-28) | 18 (13-24) | 18 (13-24) | [1,10] |
| Severe | 8 (4-14) | 10 (5-16) | 10 (6-15) | 8 (5-12) | 6 (3-10) | 6 (3-10) | [1,10] |
| Fatal | 18 (11-26) | 15 (9-22) | 27 (21-34) | 35 (28-42) | 27 (21-34) | 29 (22-36) | [1,10] |
| Haemorrhagic stroke |  | | | | | | |
| Mild | 23 (10-39) | 20 (11-30) | 35 (28-42) | 35 (28-42) | 49 (42-56) | 47 (40-54) | [1,10] |
| Moderate | 32 (17-49) | 15 (8-24) | 28 (22-35) | 22 (16-28) | 18 (13-24) | 18 (13-24) | [1,10] |
| Severe | 10 (2-23) | 12 (5-21) | 10 (6-15) | 8 (5-12) | 6 (3-10) | 6 (3-10) | [1,10] |
| Fatal | 35 (20-52) | 53 (41-65) | 27 (21-34) | 35 (28-42) | 27 (21-34) | 29 (22-36) | [1,10] |
| Haemorrhagic stroke among ICH | 77 (65-87) | 64 (55-72) | 64 (43-82) | 41 (24-59) | 57 (44-70) | 75 (63-85) | [1,12–14] |
| Patients experiencing dyspepsia | 1.67 | 1.81 | 3.69 | 3.53 | 1.67 | 1.67 | [1,12] |

^a^ The event rates of ischaemic stroke, ICH and other MB were adjusted by CHADS_2_-scores with the distribution presented in S1 Table.

Abbreviations: AC, anticoagulant; CHADS_2_, congestive heart failure, hypertension, age ≥75 years, diabetes mellitus, prior stroke or transient ischemic attack or thromboembolism; CI, confidence interval; CRNMB, clinically relevant non-major bleeding; CV, cardiovascular; GI, gastro-intestinal; HR, hazard ratio; ICH, intracranial haemorrhage; MB, major bleed; MI, myocardial infarction; NOAC, non-vitamin K antagonist oral anticoagulant; PY, patient-years; SE, systemic embolism; VKA, vitamin K antagonist.
