# Supplementary material for: Cost-effectiveness of apixaban compared to other anticoagulants in patients with atrial fibrillation in the real-world and trial settings
Source: PLoS One. 2019 Sep 17;14(9):e0222658. doi: 10.1371/journal.pone.0222658 (PMC6748426; doi:10.1371/journal.pone.0222658)
Supplement: S3 Table — Abbreviations: CI, confidence interval; HR, hazard ratio; ICH, intracranial haemorrhage; MB, major bleeding, PY, patient-years; SE, systemic embolism; VKA, vitamin K antagonist. a Hazard ratio apixaban versus comparator = (1/HR comparator versus apixaban). (DOCX) [file pone.0222658.s005.docx]

S3 Table

**Input parameters for the RWD-based analysis obtained from real-world study comparing apixaban with VKA and other NOACs by Lip et al.** [3]

| **Event** | **Event rate per 100 PY** ^a^ | | **HR (95% CI) ^b^** | | **Source** |
| --- | --- | --- | --- | --- | --- |
|  | **Apixaban** | **VKA** | **Dabigatran** | **Rivaroxaban** |  |
| Ischaemic stroke | 1.100 | 1.460 | 1.471 (1.818 -2.083) | 1.111 (0.971-1.282) | [3] |
| ICH | 0.750 | 1.450 | 0.943 (0.662-1.351) | 1.282 (1.053-1.563) | [3] |
| Other MB ^a^ | 3.260 | 5.250 | 1.190 (0.980-1.449) | 1.786 (1.613-2.000) | [3] |
| SE | 0.050 | 0.110 | 2.703 (0.862-8.333) | 2.000 (1.124-3.571) | [3] |
| Haemorrhagic stroke among ICH | 46 | 50 | 46 | 50 | [3] |
| GI bleeding among other MB | 55 | 53 | 61 | 58 | [3] |

^a^ $Hazard ratio apixaban versus comparator = (1/HR comparator versus apixaban)$

Abbreviations: CI, confidence interval; HR, hazard ratio; ICH, intracranial haemorrhage; MB, major bleeding, PY, patient-years; SE, systemic embolism; VKA, vitamin K antagonist.
