# Supplementary material for: Cost-effectiveness of apixaban compared to other anticoagulants in patients with atrial fibrillation in the real-world and trial settings
Source: PLoS One. 2019 Sep 17;14(9):e0222658. doi: 10.1371/journal.pone.0222658 (PMC6748426; doi:10.1371/journal.pone.0222658)
Supplement: S4 Table — a Lambda and Gamma are the natural logarithms of the slope of the survival hazard and age, respectively, which can be used to calculate the predicted survival at any time per age group (0–75 or >75 years) and gender. b Assumed to be equal to mortality risk adjustment factor of AF, since these events we assumed to have no additional effect on mortality risk in the period after the event. Abbreviations: AF, atrial fibrillation; CI, confidence interval; CRNMB, clinically relevant non-major bleeding; HR, hazard ratio; ICH, intracranial haemorrhage; MB, major bleeding; MI, myocardial infarction; SE, systemic embolism. (DOCX) [file pone.0222658.s006.docx]

S4 Table

**Background mortality, case fatality and mortality risk adjustment factors per event.**

| **Background mortality** | **Lambda ^a^** | **Gamma ^a^** | **Source** |
| --- | --- | --- | --- |
| Males <75 years old | -10.37 | 0.0727 | [15] |
| Males ≥75 years old | -13.97 | 0.1277 | [15] |
| Females <75 years old | -10.86 | 0.0746 | [15] |
| Females ≥75 years old | -15.36 | 0.1402 | [15] |
| **Event** | **Case fatality (%, 95% CI)** | **Additional mortality risk adjustment factors per event (HR, 95% CI)** |  |
| AF | **-** | 1.34 (1.20-1.53) | [16] |
| Stroke (ischaemic and haemorrhagic) |  |  |  |
| Mild | See Table 2 | 3.18 (1.42-4.94) | [17–19] |
| Moderate |  | 5.84 (4.08-7.60) | [17–19] |
| Severe |  | 15.75 (13.99-17.51) | [17–19] |
| Other ICH | 13 (5.9-22.3) | 1.34 (1.20-1.53) ^b^ | [1,11] |
| Other MB | 2 (1.1-3.1) | 1.34 (1.20-1.53) ^b^ | [1,11] |
| MI |  |  |  |
| Males | 10.8 (6.1-16.6) | 2.56 (2.27-2.88) | [20] |
| Females | 15.6 (8.8-24.0) | 4.16 (3.44-5.03) | [20] |
| SE | 9.4 (2.0-21.4) | 1.34 (1.20-1.53) ^b^ | [1,16] |
| CRNMB | - | 1.34 (1.20-1.53) ^b^ | [16] |

^a^ Lambda and Gamma are the natural logarithms of the slope of the survival hazard and age, respectively, which can be used to calculate the predicted survival at any time per age group (0-75 or >75 years) and gender.

^b^ Assumed to be equal to mortality risk adjustment factor of AF, since these events we assumed to have no additional effect on mortality risk in the period after the event.

Abbreviations: AF, atrial fibrillation; CI, confidence interval; CRNMB, clinically relevant non-major bleeding; HR, hazard ratio; ICH, intracranial haemorrhage; MB, major bleeding; MI, myocardial infarction; SE, systemic embolism.
