# Supplementary material for: Cost-effectiveness of apixaban compared to other anticoagulants in patients with atrial fibrillation in the real-world and trial settings
Source: PLoS One. 2019 Sep 17;14(9):e0222658. doi: 10.1371/journal.pone.0222658 (PMC6748426; doi:10.1371/journal.pone.0222658)
Supplement: S5 Table — a intracranial haemorrhage including haemorrhagic stroke. Abbreviations: CI, confidence interval; CRNMB, clinically relevant non-major bleeding; CV, cardiovascular; ICH, intracranial haemorrhage; MB, major bleeding; MI, myocardial infarction; PY, patient-years; SE, systemic embolism. (DOCX) [file pone.0222658.s007.docx]

S5 Table

**Event rates per 100 patient-years for no treatment after event unrelated treatment discontinuation.**

| **Event** | **Value (CI 95%)** | **Distribution** | **Source** |
| --- | --- | --- | --- |
| **Event rate per 100 PY** | | | |
| Ischaemic stroke | 4.186 (2.393-6.473) | Gamma | [21] |
| ICH ^a^ | 0.000 | Fixed | Assumption |
| Other MB | 0.000 | Fixed | Assumption |
| CRNMB | 0.000 | Fixed | Assumption |
| MI | 1.003 (0.573-1.551) | Gamma | [21] |
| SE | 0.959 (0.548-1.483) | Gamma | [21] |
| Other CV hospitalisation | 16.506 (9.435-25.523) | Gamma | [21] |
| **Distribution (%)** | | | |
| Haemorrhagic stroke among ICH | 46% | Fixed | [21] |
| Ischaemic stroke |  |  |  |
| Mild | 41% | Fixed | [22] |
| Moderate | 32% | Fixed | [22] |
| Severe | 5% | Fixed | [22] |
| Fatal | 22% | Fixed | [22] |

^a^ Intracranial haemorrhage including haemorrhagic stroke

Abbreviations: CI, confidence interval; CRNMB, clinically relevant non-major bleeding; CV, cardiovascular; ICH, intracranial haemorrhage; MB, major bleeding; MI, myocardial infarction; PY, patient-years; SE, systemic embolism.
