# Supplementary material for: Cost-effectiveness of apixaban compared to other anticoagulants in patients with atrial fibrillation in the real-world and trial settings
Source: PLoS One. 2019 Sep 17;14(9):e0222658. doi: 10.1371/journal.pone.0222658 (PMC6748426; doi:10.1371/journal.pone.0222658)
Supplement: S1 File — (DOCX) [file pone.0222658.s008.docx]

S1 File - Probabilistic sensitivity analysis results


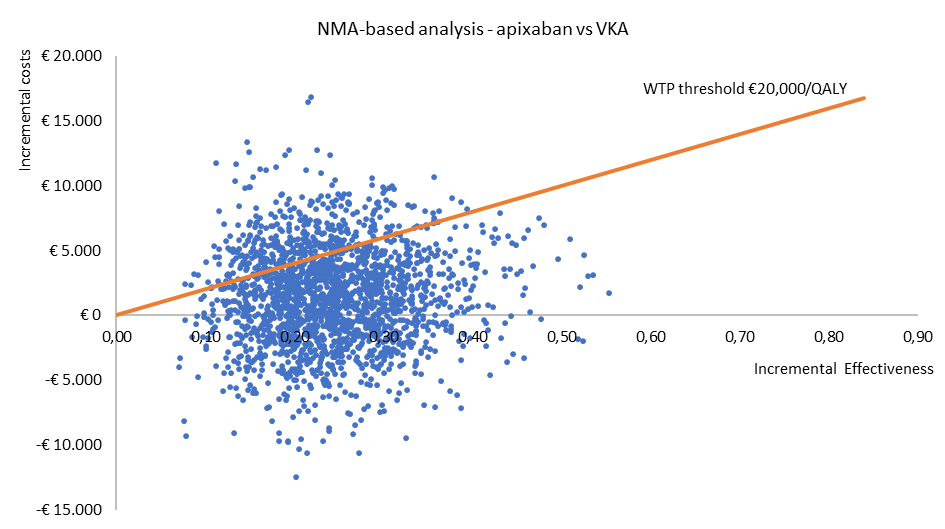


**Fig A. Probabilistic sensitivity analysis of the NMA-based analysis: apixaban versus VKA.** The red line represents the willingness-to-pay threshold of €20,000 per QALY. NMA, network meta-analysis; QALY, quality adjusted life-years; VKA, vitamin K antagonist; WTP, willingness-to-pay.


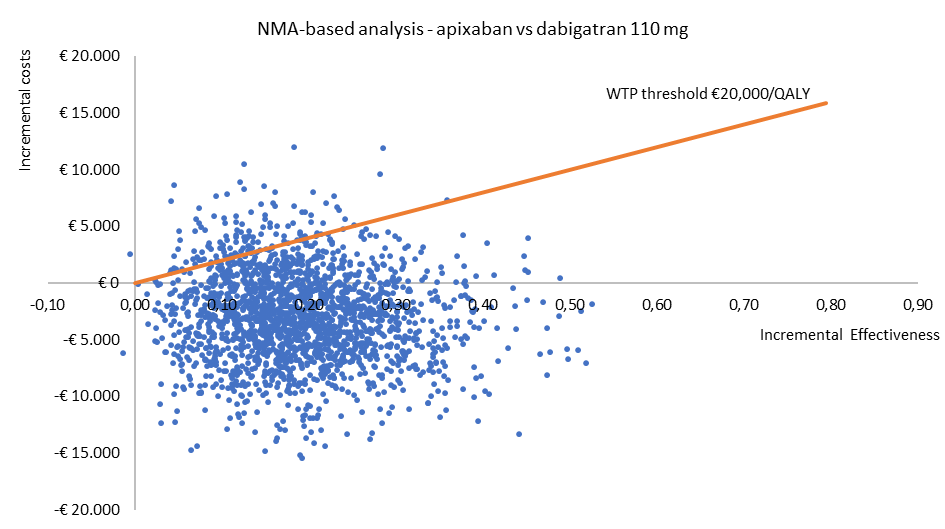


**Fig B. Probabilistic sensitivity analysis of the NMA-based analysis: apixaban versus dabigatran 110 mg.** The red line represents the willingness-to-pay threshold of €20,000 per QALY. NMA, network meta-analysis; QALY, quality adjusted life-years; WTP, willingness-to-pay.


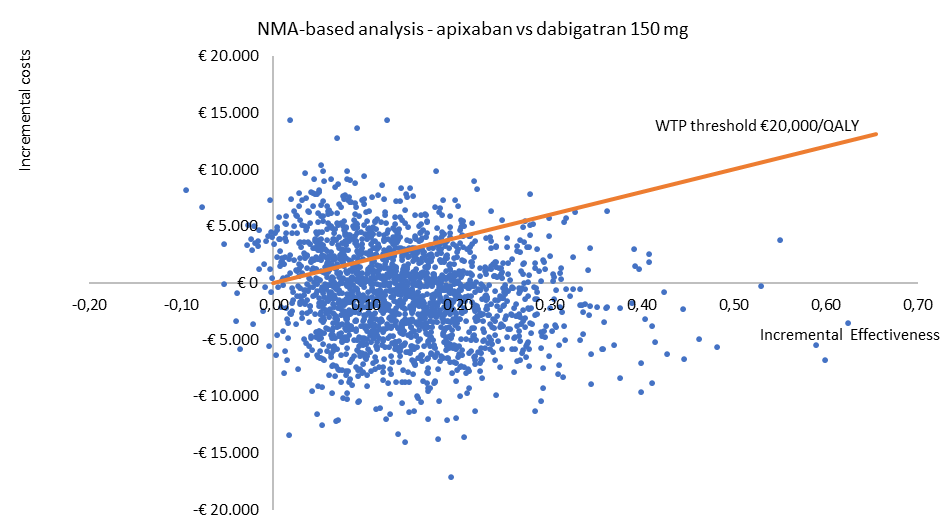


**Fig C. Probabilistic sensitivity analysis of the NMA-based analysis: apixaban versus dabigatran 150 mg.** The red line represents the willingness-to-pay threshold of €20,000 per QALY. NMA, network meta-analysis; QALY, quality adjusted life-years; WTP, willingness-to-pay.


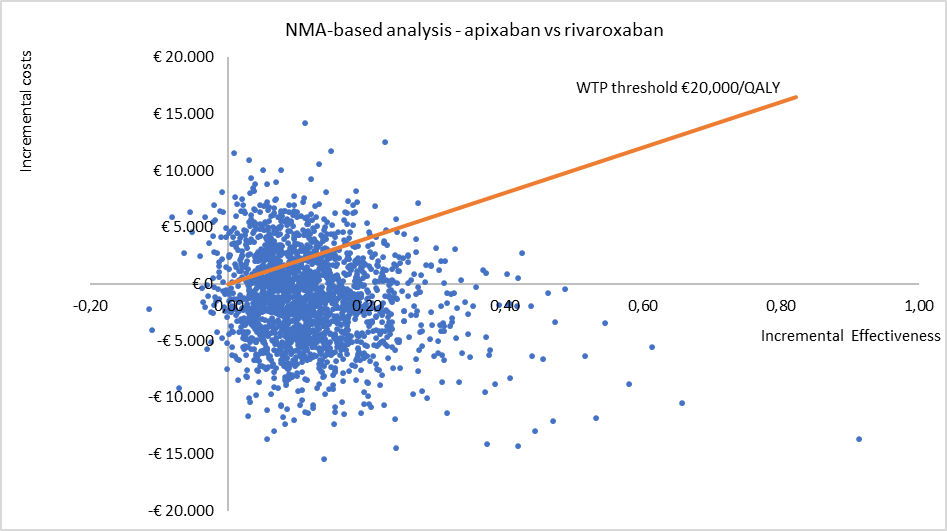


**Fig D. Probabilistic sensitivity analysis of the NMA-based analysis: apixaban versus rivaroxaban.** The red line represents the willingness-to-pay threshold of €20,000 per QALY. NMA, network meta-analysis; QALY, quality adjusted life-years; WTP, willingness-to-pay.


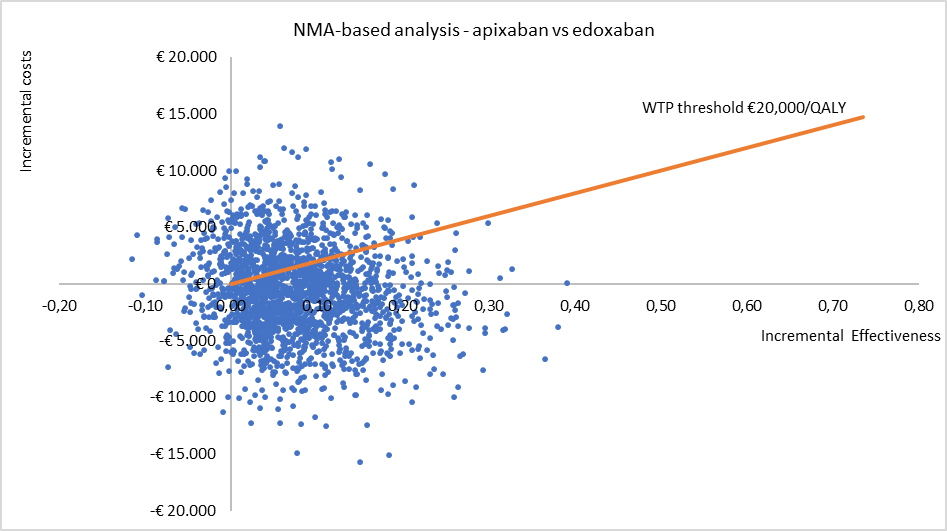


**Fig E. Probabilistic sensitivity analysis of the NMA-based analysis: apixaban versus edoxaban.** The red line represents the willingness-to-pay threshold of €20,000 per QALY. NMA, network meta-analysis; QALY, quality adjusted life-years; WTP, willingness-to-pay.


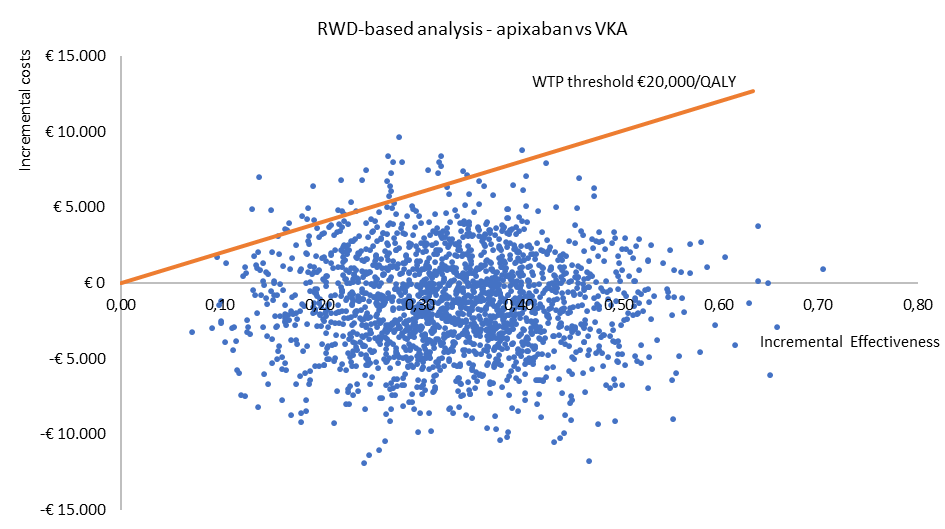


**Fig F. Probabilistic sensitivity analysis of the RWD-based analysis: apixaban versus VKA.** The red line represents the willingness-to-pay threshold of €20,000 per QALY. QALY, quality adjusted life-years; RWD, real-world data; VKA, vitamin K antagonist; WTP, willingness-to-pay.

**
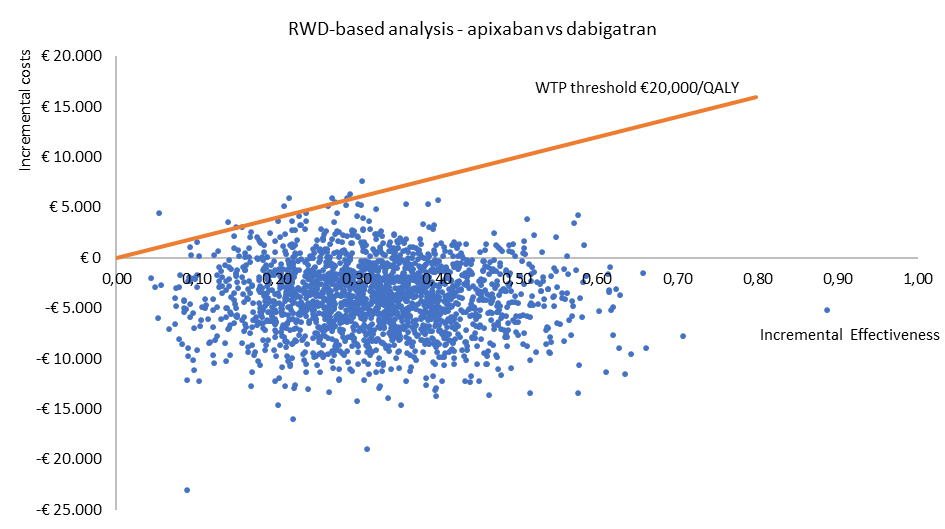
**

**Fig G.** **Probabilistic sensitivity analysis of the RWD-based analysis: apixaban versus dabigatran.** The red line represents the willingness-to-pay threshold of €20,000 per QALY. QALY, quality adjusted life-years; RWD, real-world data; WTP, willingness-to-pay.


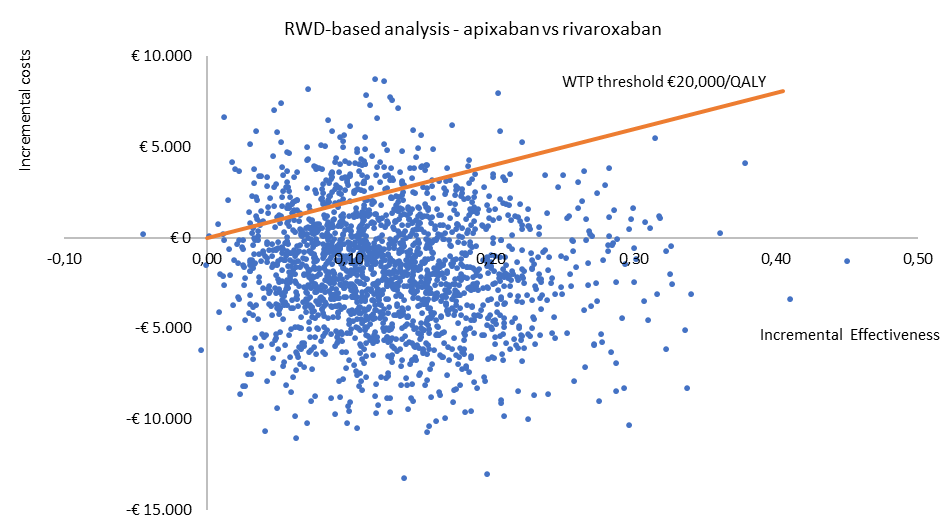


**Fig H.** **Probabilistic sensitivity analysis of the RWD-based analysis: apixaban versus rivaroxaban.** The red line represents the willingness-to-pay threshold of €20,000 per QALY. QALY, quality adjusted life-years; RWD, real-world data; WTP, willingness-to-pay.
