# Supplementary material for: Correction: Cost-effectiveness of apixaban compared to other anticoagulants in patients with atrial fibrillation in the real-world and trial settings
Source: PLoS One. 2022 Mar 31;17(3):e0266625. doi: 10.1371/journal.pone.0266625 (PMC8970364; doi:10.1371/journal.pone.0266625)
Supplement: S1 Table — (DOCX) [file pone.0266625.s001.docx]

S1 Table

**Patient baseline characteristics model inputs used in the NMA-based and RWD-based analyses.**

| **Characteristic** | **Value (mean)** | **Reference** |
| --- | --- | --- |
| **NMA-based analysis** | | |
| Age |  |  |
| Male (years) | 71.5 | [1] |
| Female (years) | 71.5 | [1] |
| Gender (male, %) | 64.7 | [1] |
| CHADS_2_ distribution |  |  |
| 0-1 (%) | 50.7 | [1] |
| 2 (%) | 29.1 | [1] |
| >3 (%) | 20.2 | [1] |
| Average CHADS_2_ score | 1.7 | [1] |
| Average CHA_2_DS_2_-VASc score | 3.1 | [1] |
| **RWD-based analysis** | | |
| Age |  |  |
| Male (years) | 74.3 | [2,3] |
| Female (years) | 74.3 | [2,3] |
| Gender (male, %) | 54.1 | [2,3] |
| CHA_2_DS_2_-VASc score distribution |  |  |
| 0-1 (%) | 9.8 | [2,3] |
| 2 (%) | 14.5 | [2,3] |
| >3 (%) | 75.7 | [2,3] |
| Average CHA_2_DS_2_-VASc score | 3.7 | [2,3] |

Abbreviations: CHADS_2_ score, congestive heart failure, hypertension, age ≥75 years, diabetes mellitus, prior stroke or transient ischemic attack or thromboembolism; CHA_2_DS_2_-VASc score, congestive heart failure, hypertension, age ≥75 years (2), diabetes mellitus, prior stroke or transient ischemic attack or thromboembolism (2) and vascular disease (peripheral arterial disease, previous MI, aortic atheroma)

**References**

1. Korenstra J, Petra E, Wijtvliet J, Veeger NJGM, Geluk CA, Bartels GL, et al. Effectiveness and safety of dabigatran versus acenocoumarol in “real-world” patients with atrial fibrillation. Europace. 2016;18:1319–27.

2. Lip GY, Keshishian A, Li X, Hamilton M, Masseria C, Gupta K, et al. Effectiveness and Safety of Oral Anticoagulants Among Nonvalvular Atrial Fibrillation Patients. Stroke. 2018;49(0):00.

3. Correction to: Effectiveness and Safety of Oral Anticoagulants Among Nonvalvular Atrial Fibrillation Patients: The ARISTOPHANES Study. Stroke. 2020 Jan 10;
