# Supplementary material for: Correction: Cost-effectiveness of apixaban compared to other anticoagulants in patients with atrial fibrillation in the real-world and trial settings
Source: PLoS One. 2022 Mar 31;17(3):e0266625. doi: 10.1371/journal.pone.0266625 (PMC8970364; doi:10.1371/journal.pone.0266625)
Supplement: S3 Table — (DOCX) [file pone.0266625.s003.docx]

S3 Table

**Input parameters for the RWD-based analysis obtained from real-world study comparing apixaban with VKA and other NOACs by Lip et al.** [1]

| **Event** | **Event rate per 100 PY** | | **HR (95% CI) ^c^** | | **Source** |
| --- | --- | --- | --- | --- | --- |
|  | **Apixaban** | **VKA** | **Dabigatran** | **Rivaroxaban** |  |
| Ischaemic stroke | 1.050 | 1.320 | 1.449 (1.190 -1.754) | 1.176 (1.053-1.316) | [1,2] |
| ICH  ^a^ | 0.740 | 1.390 | 0.962 (0.704-1.316) | 1.163 (1.000-1.370) | [1,2] |
| Other MB ^b^ | 3.320 | 5.060 | 1.136 (0.962-1.351) | 1.754 (1.639-1.923) | [1,2] |
| SE | 0.050 | 0.100 | 2.273 (0.943-5.556) | 2.128 (1.351-3.448) | [1,2] |
| Haemorrhagic stroke among ICH | 32 | 37 | 29 | 35 | [1,2] |
| GI bleeding among other MB | 53 | 53 | 56 | 53 | [1,2] |

^a^ Composite of ICH and haemorrhagic stroke assuming censoring; ^b^ Composite of other bleeding and GI bleeding assuming censoring; ^c^ $Hazard ratio apixaban versus comparator = (1/HR comparator versus apixaban)$

Abbreviations: CI, confidence interval; HR, hazard ratio; ICH, intracranial haemorrhage; MB, major bleeding, PY, patient-years; SE, systemic embolism; VKA, vitamin K antagonist.

**References**

1. Lip GY, Keshishian A, Li X, Hamilton M, Masseria C, Gupta K, et al. Effectiveness and Safety of Oral Anticoagulants Among Nonvalvular Atrial Fibrillation Patients. Stroke. 2018;49(0):00.

2. Correction to: Effectiveness and Safety of Oral Anticoagulants Among Nonvalvular Atrial Fibrillation Patients: The ARISTOPHANES Study. Stroke. 2020 Jan 10;
