# Supplementary material for: Correction: Cost-effectiveness of apixaban compared to other anticoagulants in patients with atrial fibrillation in the real-world and trial settings
Source: PLoS One. 2022 Mar 31;17(3):e0266625. doi: 10.1371/journal.pone.0266625 (PMC8970364; doi:10.1371/journal.pone.0266625)
Supplement: S4 Table — (DOCX) [file pone.0266625.s004.docx]

S4 Table

**Background mortality, case fatality and mortality risk adjustment factors per event.**

| **Background mortality** | **Lambda ^a^** | **Gamma ^a^** | **Source** |
| --- | --- | --- | --- |
| Males <75 years old | -10.37 | 0.0727 | [1] |
| Males ≥75 years old | -13.97 | 0.1277 | [1] |
| Females <75 years old | -10.86 | 0.0746 | [1] |
| Females ≥75 years old | -15.36 | 0.1402 | [1] |
| **Event** | **Case fatality (%, 95% CI)** | **Additional mortality risk adjustment factors per event (HR, 95% CI)** |  |
| AF | **-** | 1.34 (1.20-1.53) | [2] |
| Stroke (ischaemic and haemorrhagic) |  |  |  |
| Mild | See Table 2 | 3.18 (1.42-4.94) | [3–5] |
| Moderate |  | 5.84 (4.08-7.60) | [3–5] |
| Severe |  | 15.75 (13.99-17.51) | [3–5] |
| Other ICH | 13 (5.9-22.3) | 1.34 (1.20-1.53) ^b^ | [6,7] |
| Other MB | 2 (1.1-3.1) | 1.34 (1.20-1.53) ^b^ | [6,7] |
| MI |  |  |  |
| Males | 10.8 (6.1-16.6) | 2.56 (2.27-2.88) | [8] |
| Females | 15.6 (8.8-24.0) | 4.16 (3.44-5.03) | [8] |
| SE | 9.4 (2.0-21.4) | 1.34 (1.20-1.53) ^b^ | [2,7] |
| CRNMB | - | 1.34 (1.20-1.53) ^b^ | [2] |

^a^ Lambda and Gamma are the natural logarithms of the slope of the survival hazard and age, respectively, which can be used to calculate the predicted survival at any time per age group (0-75 or >75 years) and gender.

^b^ Assumed to be equal to mortality risk adjustment factor of AF, since these events we assumed to have no additional effect on mortality risk in the period after the event.

Abbreviations: AF, atrial fibrillation; CI, confidence interval; CRNMB, clinically relevant non-major bleeding; HR, hazard ratio; ICH, intracranial haemorrhage; MB, major bleeding; MI, myocardial infarction; SE, systemic embolism.

**References**

1. Statistics Netherlands (CBS). Life expectancy; sex, age (per year and period of five years) [Internet].

2. Friberg L, Hammar N, Pettersson H, Rosenqvist M. Increased mortality in paroxysmal atrial fibrillation: report from the Stockholm Cohort-Study of Atrial Fibrillation (SCAF). Eur Heart J. 2007;28(19):2346–53.

3. Brønnum-Hansen H, Davidsen M, Thorvaldsen P, Danish MONICA Study Group. Long-term survival and causes of death after stroke. Stroke. 2001;32(9):2131–6.

4. Henriksson KM, Farahmand B, Johansson S, Asberg S, Terént A, Edvardsson N. Survival after stroke--the impact of CHADS2 score and atrial fibrillation. Int J Cardiol. 2010;141(1):18–23.

5. Huybrechts KF, Caro JJ, Xenakis JJ, Vemmos KN. The prognostic value of the modified Rankin Scale score for long-term survival after first-ever stroke. Results from the Athens Stroke Registry. Cerebrovasc Dis. 2008;26(4):381–7.

6. Connolly SJ, Eikelboom J, Joyner C, Diener H-C, Hart R, Golitsyn S, et al. Apixaban in Patients with Atrial Fibrillation. N Engl J Med. 2011;364(9):806–17.

7. Granger CB, Alexander JH, McMurray JJV, Lopes RD, Hylek EM, Hanna M, et al. Apixaban versus Warfarin in Patients with Atrial Fibrillation. N Engl J Med. 2011;365(11):981–92.

8. Brønnum-Hansen H, Jørgensen T, Davidsen M, Madsen M, Osler M, Gerdes LU, et al. Survival and cause of death after myocardial infarction: the Danish MONICA study. J Clin Epidemiol. 2001;54(12):1244–50.
