# Supplementary material for: Correction: Cost-effectiveness of apixaban compared to other anticoagulants in patients with atrial fibrillation in the real-world and trial settings
Source: PLoS One. 2022 Mar 31;17(3):e0266625. doi: 10.1371/journal.pone.0266625 (PMC8970364; doi:10.1371/journal.pone.0266625)
Supplement: S5 Table — (DOCX) [file pone.0266625.s005.docx]

S5 Table

**Event rates per 100 patient-years for no treatment after event unrelated treatment discontinuation.**

| **Event** | **Value (CI 95%)** | **Distribution** | **Source** |
| --- | --- | --- | --- |
| **Event rate per 100 PY** | | | |
| Ischaemic stroke | 4.186 (2.393-6.473) | Gamma | [1] |
| ICH ^a^ | 0.000 | Fixed | Assumption |
| Other MB | 0.000 | Fixed | Assumption |
| CRNMB | 0.000 | Fixed | Assumption |
| MI | 1.003 (0.573-1.551) | Gamma | [1] |
| SE | 0.959 (0.548-1.483) | Gamma | [1] |
| Other CV hospitalisation | 16.506 (9.435-25.523) | Gamma | [1] |
| **Distribution (%)** | | | |
| Haemorrhagic stroke among ICH | 46% | Fixed | [1] |
| Ischaemic stroke |  |  |  |
| Mild | 41% | Fixed | [2] |
| Moderate | 32% | Fixed | [2] |
| Severe | 5% | Fixed | [2] |
| Fatal | 22% | Fixed | [2] |

^a^ Intracranial haemorrhage including haemorrhagic stroke

Abbreviations: CI, confidence interval; CRNMB, clinically relevant non-major bleeding; CV, cardiovascular; ICH, intracranial haemorrhage; MB, major bleeding; MI, myocardial infarction; PY, patient-years; SE, systemic embolism.

**References**

1. Mandema J. Meta analysis of placebo, ASA and warfarin controlled studies in AF. Data file. 2011;

2. Hylek EM, Go AS, Chang Y, Jensvold NG, Henault LE, Selby J V., et al. Effect of Intensity of Oral Anticoagulation on Stroke Severity and Mortality in Atrial Fibrillation. N Engl J Med. 2003;349(11):1019–26.
